# Berberine signature and cardiometabolic diseases using randomized controlled trial, cohort study and Mendelian randomization

**DOI:** 10.1038/s44325-026-00113-w

**Published:** 2026-03-25

**Authors:** Jie V. Zhao, Vishal Sarsani, Bing Chen, Huan Yun, Jie Hu, Liming Liang

**Affiliations:** 1 School of Public Health, Li Ka Shing Faculty of Medsicine, the University of Hong Kong, Hong Kong, SAR China; 2 Department of Epidemiology, Harvard T.H. Chan School of Public Health, Boston, MA USA; 3 Center for Genomic Medicine and Department of Anesthesia, Critical Care and Pain Medicine, Massachusetts General Hospital and Harvard Medical School, Boston, MA USA; 4 Department of Biostatistics, Harvard T.H. Chan School of Public Health, Boston, MA USA

**Keywords:** Cardiovascular diseases, Endocrine system and metabolic diseases

## Abstract

Berberine lowers both lipids and glucose, yet its role on cardiometabolic disease risk remain unclear. Based on a randomized controlled trial of berberine (registered in ClinicalTrials.gov on Dec 2018, NCT03770325), leveraging proteomics and sex hormones data, we built a signature reflecting response to berberine using elastic net regression. We then assessed its associations with ischemic heart disease (IHD) and diabetes in UK Biobank using logistic regression and for causal relationship, using bi-directional Mendelian randomization (MR), and estimates of each protein/hormone. The signature was related to lower IHD and diabetes risks (OR for IHD 0.85, 95% CI 0.79-0.91; diabetes 0.88, 0.80-0.96 using MR). SHBG and PRSS2 might explain the beneficial association with IHD; testosterone, SHBG, CCL5, CNDP1, F11, LCN2, and THBS4 might explain the association with diabetes, which provides insights for drug development. Our study suggests beneficial associations of berberine with IHD and diabetes, which requires confirmation in large clinical trials.

## Introduction

Ischemic heart disease (IHD) is the single leading cause of mortality and poses a heavy burden on healthcare^1^, which accounts for ~16% of all deaths globally^2^. Hyperlipidemia is one of the most important risk factors for IHD. Lipid lowering therapy has been one of the main treatments for IHD. Since the discovery of statins in Japan in the early 1970s, statins have been the mainstay for the treatment of hyperlipidemia^3^. However, statins are not tolerable for everyone because of adverse events such as myalgia and hyperglycemia^4^. As such, other lipid-lowering drugs are needed as alternatives or to be used in combination with statins. Proprotein convertase subtilisin/kexin type 9 (PCSK9) inhibitors are a newer class of lipid-lowering drugs, but they are expensive and may not be accessible to all who need it.

Berberine has got arising attention in recent years due to its lipid-lowering effect^5^. In contrast to statins’ pleiotropic effect on diabetes and hyperglycemia^6^, berberine improves glucose metabolism^7^. As such, it may be beneficial to use berberine combined with statins in people with hyperlipidemia, especially for those with statin intolerance or partial intolerance, and those with diabetes or at high risk of diabetes. Berberine has been recommended by the International Lipid Expert Panel and the 2019 European Atherosclerosis Society/European Society of Cardiology Guidelines for the treatment of hyperlipidemia in statin-intolerant patients^8^, however, these guidelines have not provided explicit recommendations about the use of berberine because of the lack of high-quality evidence. Despite of the benefits on lipid profile and glucose metabolism, the effect of berberine on the risk of IHD and diabetes has not been clarified. A large randomized controlled trial (RCT) may take years and need very large expenses. To provide insights to this important question prior to conducting a large RCT, an alternative way is to examine the effect of the response to berberine, proxied by the intermediate biomarkers, such as proteins. This method has been applied previously to examine the effect of Mediterranean diet on cardiovascular disease^9^. Proteins play an important role in metabolism. Recent advances in high-throughput proteomics profiling provide a valuable platform to measure multiple proteins at one go^10^.

We previously conducted an RCT of berberine in men to examine the effects of berberine on cardiovascular disease risk factors, including lipids and glucose metabolism^7,11^. Using the stored blood samples, we also measured proteins that can be used to build response to berberine. In the trial, we focused on men, because men have a higher risk of IHD, and berberine may have a sex-specific effect^7^. It is increasingly recognized that men and women have differential responses to medications metabolized by uridine-glucuronosyltransferase (UGT) enzymes, possibly due to the role of sex hormones^12^. UGT enzymes are also involved in the metabolism of berberine^13^. Berberine might also be a driver of sex hormones. As shown in our completed trial^11^ and previous studies^14,15^, berberine increased serum total testosterone in men^11^ and increased sex hormone binding globulin (SHBG) in men and women^14,15^. As such, considering the potential sex disparity is necessary when assessing the effects of berberine.

**Fig. 1:**
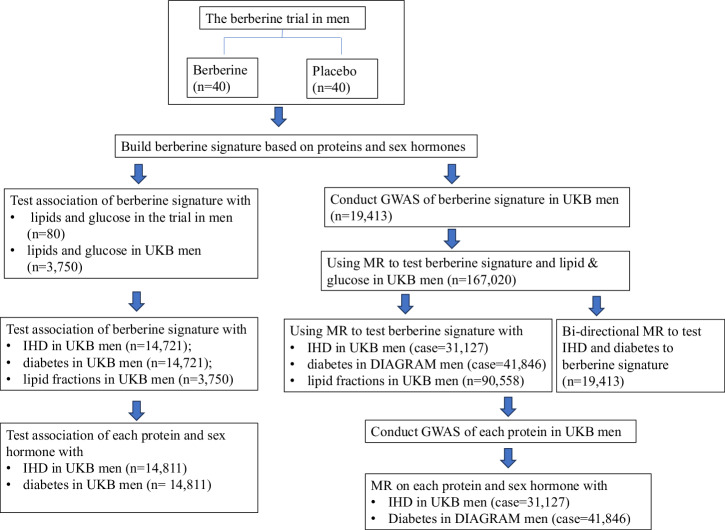
Flow chart of the study design. The specific steps and study flow were shown in this figure.

Taking advantage of the berberine trial and UK Biobank, a large cohort with proteomics and comprehensive measures of disease outcomes, we examined the association of berberine signature, built using our berberine trial, with the risk of IHD and diabetes using conventional observational study in men in UK Biobank based on the proteomics and sex hormones data (for short “UKB proteomics study”), and tested potential causality using Mendelian randomization (MR) (Fig. 1), a robust analysis which used genetic variants as instrument to avoid environment confounding for casual inference^16^.

## Results

### The berberine signature and its association with lipids and glucose

The elastic net regression selected 18 proteins and 2 sex hormones. The area under the curve based on leave-one-out cross validation is 0.61 (Supplementary Fig. 1). The berberine signature was built as follows:

testosterone*0.21 + SHBG*0.21 + CA1*0.007 + CCL5*−0.23 + LTBP2*−0.14 + F11*−0.13 + COMP*-0.004 + CA4*0.08 + MBL2*0.04 + CD46*0.01 + REG1A*0.27 + FCN2*−0.03 + PRSS2*-0.35 + CNDP1*−0.18 + THBS4*−0.15 + TGFBR3*−0.11 + DEFA1*0.27 + APOM*−0.25 + LCN2*−0.025 + PLA2G7*−0.14

Permutation analysis showed that the coefficients of the selected proteins were consistently larger than those obtained under random assignment, with empirical p-values below 0.05 (Supplementary Fig. 2), indicating that the observed associations are not driven by random variation. In the cross-validation, nearly all proteins were selected in most folds, and in every instance their coefficient directions were consistent with those observed in the signature (Supplementary Table 1). CA1 was less stable; however, its coefficient in the signature was close to zero (0.007).

Using GWAS, we identified 1040 SNPs reaching genome-wide significance. The Manhattan plot was shown in Supplementary Fig. 3. After clumping at *r*^2^ of 0.01, we identified 7 SNPs mapped to genes *SHBG*, *SERPINA1*, *GCKR*, *ZBTB4*, *CHRDL1* and *JMJD1C* (Supplementary Table 2) and used them in the following MR analyses to examine the effect of berberine signature on lipids, glucose, IHD, diabetes and lipid fractions.

When assessing the association of berberine signature with lipids and glucose in the trial and in UK Biobank, as expected, we found the berberine signature showed an inverse association with total cholesterol, fasting glucose and LDL-cholesterol in UK Biobank using linear regression and MR analyses. The berberine signature also showed the trend of lowering total cholesterol, fasting glucose and LDL-cholesterol in the trial, although the associations were not statistically significant, possibly due to the relatively small sample size of the trial (Table 1).

**Table 1:** The effect of berberine on lipids and glucose, the association of berberine signature with lipids and glucose in the berberine trial and in UK Biobank using observational study and Mendelian randomization

Study design	Exposure	Data source	Outcome (SD)	beta	95% CI	*P* value
RCT	Berberine treatment	Berberine trial	Total cholesterol	−0.45	−0.88, −0.02	0.04
			Fasting glucose	−0.59	−1.02, −0.17	0.007
			LDL-cholesterol	−0.39	−0.83, 0.04	0.08
Observational association	Berberine signature	Berberine trial	Total cholesterol	−0.21	−0.78, 0.36	0.47
			Fasting glucose	−0.34	−0.89, 0.21	0.23
			LDL-cholesterol	−0.05	−0.62, 0.52	0.85
Observational association^a^	Berberine signature	UK Biobank	Total cholesterol	−0.27	−0.31, −0.23	<2 × 10^−16^
			Fasting glucose	−0.08	−0.12, −0.04	2.2 × 10^−6^
			LDL-cholesterol	−0.30	−0.34, −0.26	<2 × 10^−16^
Mendelian randomization^b^	Genetically predicted berberine signature	UK Biobank	Total cholesterol	−0.07	−0.11, −0.04	1.0 × 10^−4^
			Fasting glucose	−0.04	−0.07, 0.00	0.06
			LDL-cholesterol	−0.06	−0.09, −0.02	0.002

^a^Adjusted for age, ethnicity, smoking, alcohol drinking, Townsend index, education, and physical activity.

^b^The estimates are from weighted median, which is more robust to pleiotropy.

### The association of berberine signature with IHD and diabetes in UKB proteomics study and MR

Using multivariable logistic regression, we found berberine signature was associated with lower risk of IHD and diabetes after controlling for multiple confounders (Table 2). The association was supported by one-sample MR analysis (Table 2). The associations were also corroborated by using different MR analytic methods (Table 3). We excluded one SNP (rs5985544) for IHD, a potentially pleiotropic SNP indicated by MR-PRESSO, and one SNP (rs780093) for diabetes as a potentially pleiotropic SNP indicated by scatter plot (Supplementary Fig. 4) and MR-PRESSO. In the bi-directional MR analysis on the effect of IHD and diabetes on berberine signature, based on 41 SNPs for IHD (Supplementary Table 3) and 118 SNPs for diabetes (Supplementary Table 4), we found no evidence that IHD and diabetes affect berberine signature (Table 3). MR-Egger intercept did not indicate directional pleiotropy (Table 3).

**Table 2:** The association of berberine signature with ischemic heart disease and diabetes in UK Biobank using logistic regression and using one-sample Mendelian randomization

Data source	Outcomes	OR	95% CI	*P* value
Observational association^a^	IHD	0.87	0.80, 0.94	5.0 × 10^−4^
	Diabetes	0.66	0.60, 0.72	<2 × 10^−16^
Mendelian randomization	IHD	0.85	0.79, 0.91	1.6 × 10^−5^
	Diabetes	0.88	0.80, 0.96	0.006

^a^Adjusted for age, ethnicity, smoking, alcohol drinking, Townsend index, education, and physical activity.

**Table 3:** The bi-directional association of berberine signature with ischemic heart disease and diabetes using different two-sample Mendelian randomization methods (IVW, weighted median, weighted mode, MR-PRESSO, MR-Egger, GSMR)

Exposure	Outcome	#SNPs	Methods	OR	95% CI	*p*	MR-Egger intercept p
Berberine signature	IHD	6^a^	IVW	0.83	0.74, 0.94	0.003	
			Weighted median	0.86	0.79, 0.95	0.002	
			Weighted mode	0.87	0.79, 0.95	0.003	
			MR-PRESSO	0.83	0.74, 0.94	0.03	
			MR-Egger	0.90	0.70, 1.15	0.40	0.474
			GSMR	0.84	0.78, 0.91	2.50 × 10^−5^	
	Diabetes	6	IVW	0.88	0.80, 0.97	0.008	
			Weighted median	0.90	0.80, 1.01	0.068	
			Weighted mode	0.91	0.80, 1.03	0.137	
			MR-PRESSO	0.88	0.80, 0.96	0.036	
			MR-Egger	0.99	0.84, 1.16	0.863	0.100
			GSMR	0.88	0.80, 0.97	0.011	
IHD	Berberine signature	41	IVW	−0.01	−0.06, 0.03	0.513	
			Weighted median	−0.02	−0.08, 0.04	0.545	
			Weighted mode	0.01	−0.07, 0.08	0.852	
			MR-PRESSO	−0.01	−0.06, 0.03	0.517	
			MR-Egger	0.02	−0.08, 0.11	0.75	0.505
			GSMR	−0.02	−0.06, 0.02	0.29	
Diabetes	Berberine signature	119	IVW	−0.02	−0.05, 0.02	0.34	
			Weighted median	−0.002	−0.04, 0.04	0.93	
			Weighted mode	0.03	−0.02, 0.08	0.28	
			MR-PRESSO^b^	−0.01	−0.04, 0.02	0.56	
			MR-Egger	0.04	−0.04, 0.11	0.31	0.106
			GSMR	−0.02	−0.04, 0.006	0.15	

^a^One SNP (rs5985544) was excluded for IHD, a potentially pleiotropic SNP indicated by MR-PRESSO, and one SNP (rs780093) for diabetes, a potentially pleiotropic SNP indicated by scatter plot (Supplementary Fig. 4) and MR-PRESSO.

^b^One SNP (rs1260326) was excluded in the analysis on genetically predicted diabetes and berberine signature.

### The association of berberine signature with lipid fractions in UKB proteomics study and MR

**Fig. 2:**
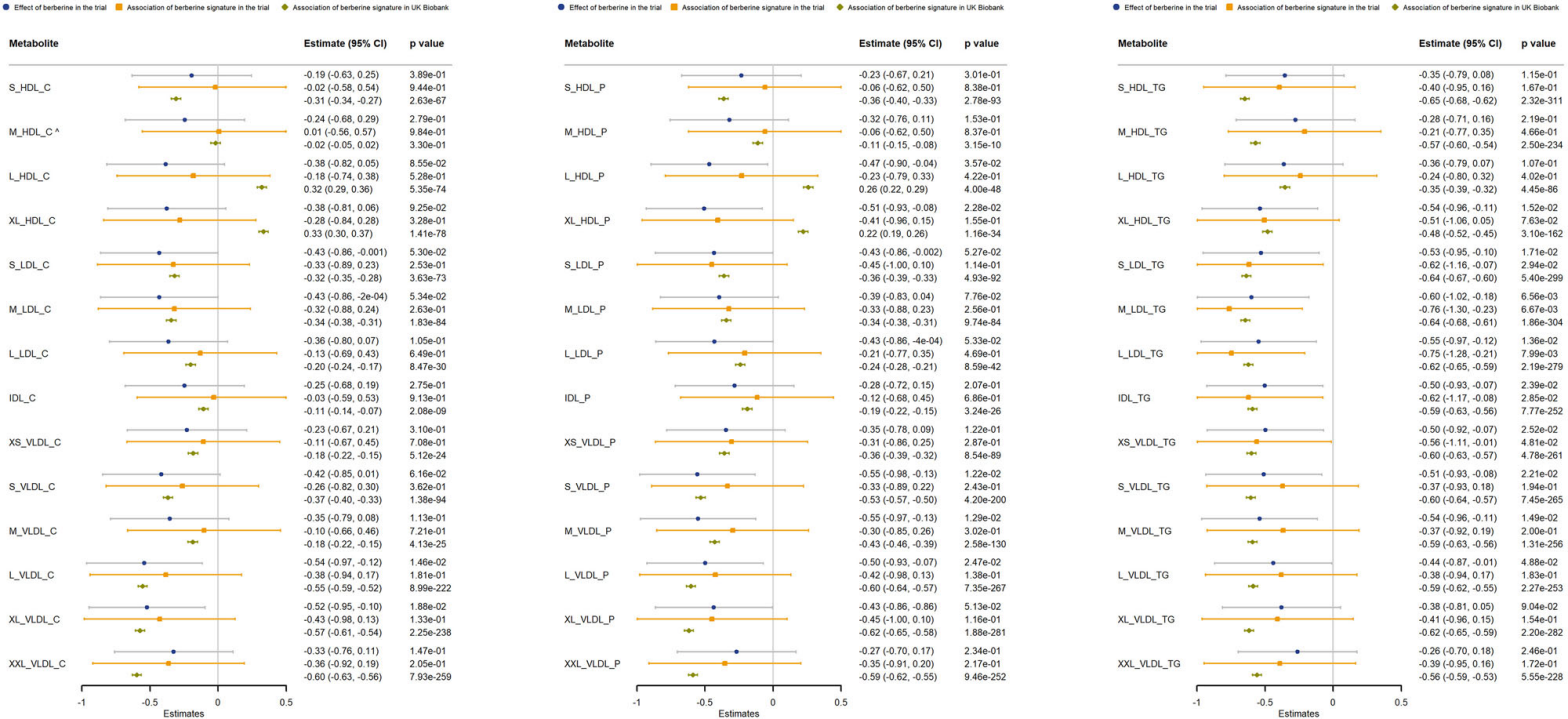
The effect of berberine on lipid fractions, and associations of berberine signature with lipid fractions in berberine trial in 80 men and in UK Biobank in 3750 men. Multivariable linear regression was used to obtain the associations of berberine signature with lipid fractions. The symbol (^) means that the association was not statistically significant after correction for multiple testing at FDR < 0.05 in UK Biobank.

**Fig. 3:**
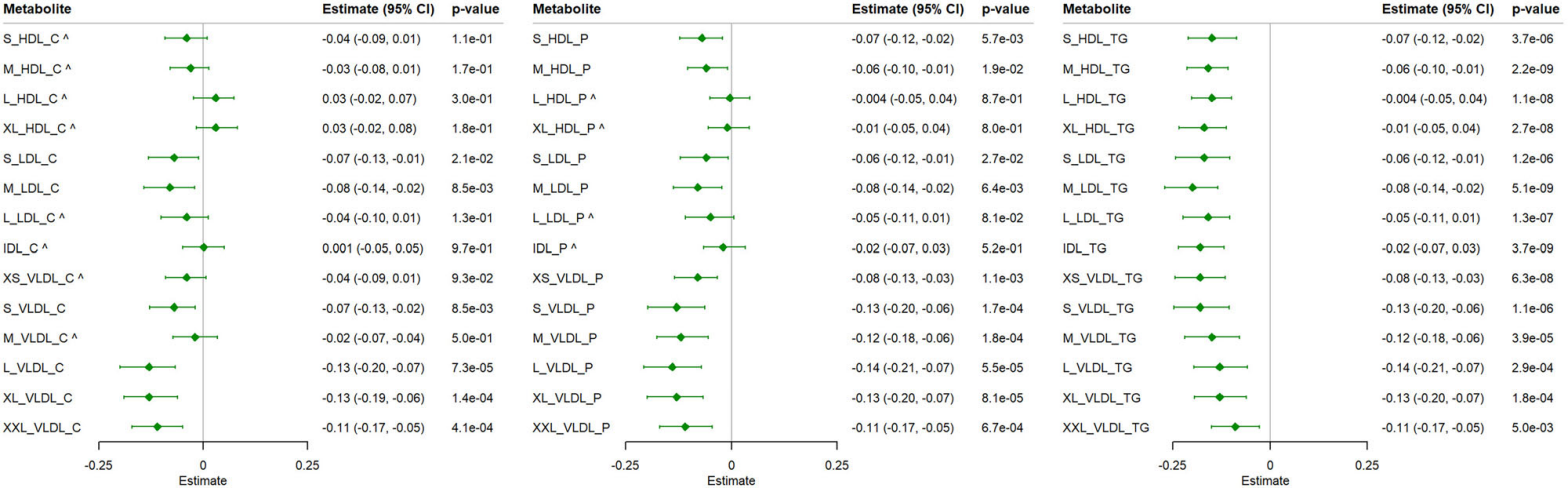
Mendelian Randomization (MR) analyses on the associations of berberine signature with lipid fractions in 90,558 men in UK Biobank. Estimates from weighted median, a method more robust to pleiotropy, were plotted. The symbol (^^^) means that the association was not statistically significant after correction for multiple testing at FDR < 0.05.

In the further analyses on lipid fractions, we compared the estimates of berberine on lipid fractions in the trial, the association of berberine signature with lipid fractions using linear regression and MR analyses (Figs. 2 and 3). Among the 42 lipid fractions, we identified several that reached statistical significance after correction for multiple testing (FDR < 0.05) in both UK Biobank linear regression (Fig. 2) and MR analyses (Fig. 3), and with consistent directions in the berberine trial (Fig. 2). Specifically, we found that the berberine signature was associated with lower cholesterol in large to extremely large VLDL, small VLDL, and small to medium LDL, lower particle concentrations across VLDL, small to medium LDL and HDL, and lower triglycerides across VLDL, LDL, HDL, and IDL subclasses. The associations were corroborated by MR analyses using different MR analytic methods (Fig. 3 and Supplementary Fig. 5) on the associations of berberine signature with these lipid fractions in men in UK Biobank.

### The association of individual protein and sex hormone in the berberine signature with IHD and diabetes in UKB proteomics study and MR

**Fig. 4:**
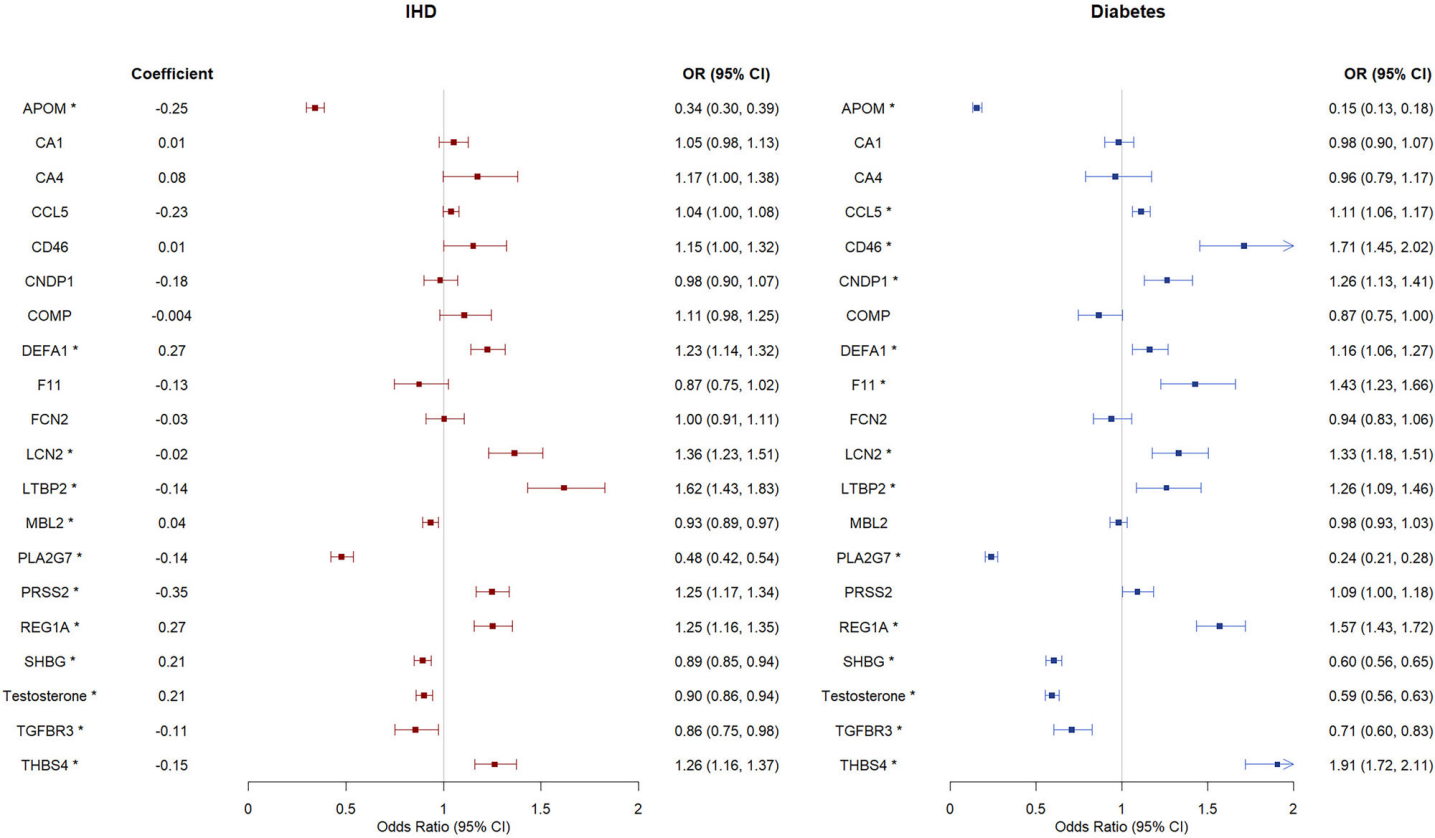
The associations of each protein/sex hormone in berberine signature with ischemic heart disease (IHD) and diabetes in observational study among 14,721 men in UK Biobank. Multivariable logistic regression was used to obtain the associations. The symbol (^*^) means that the association was statistically significant after correction for multiple testing at FDR < 0.05.

**Fig. 5:**
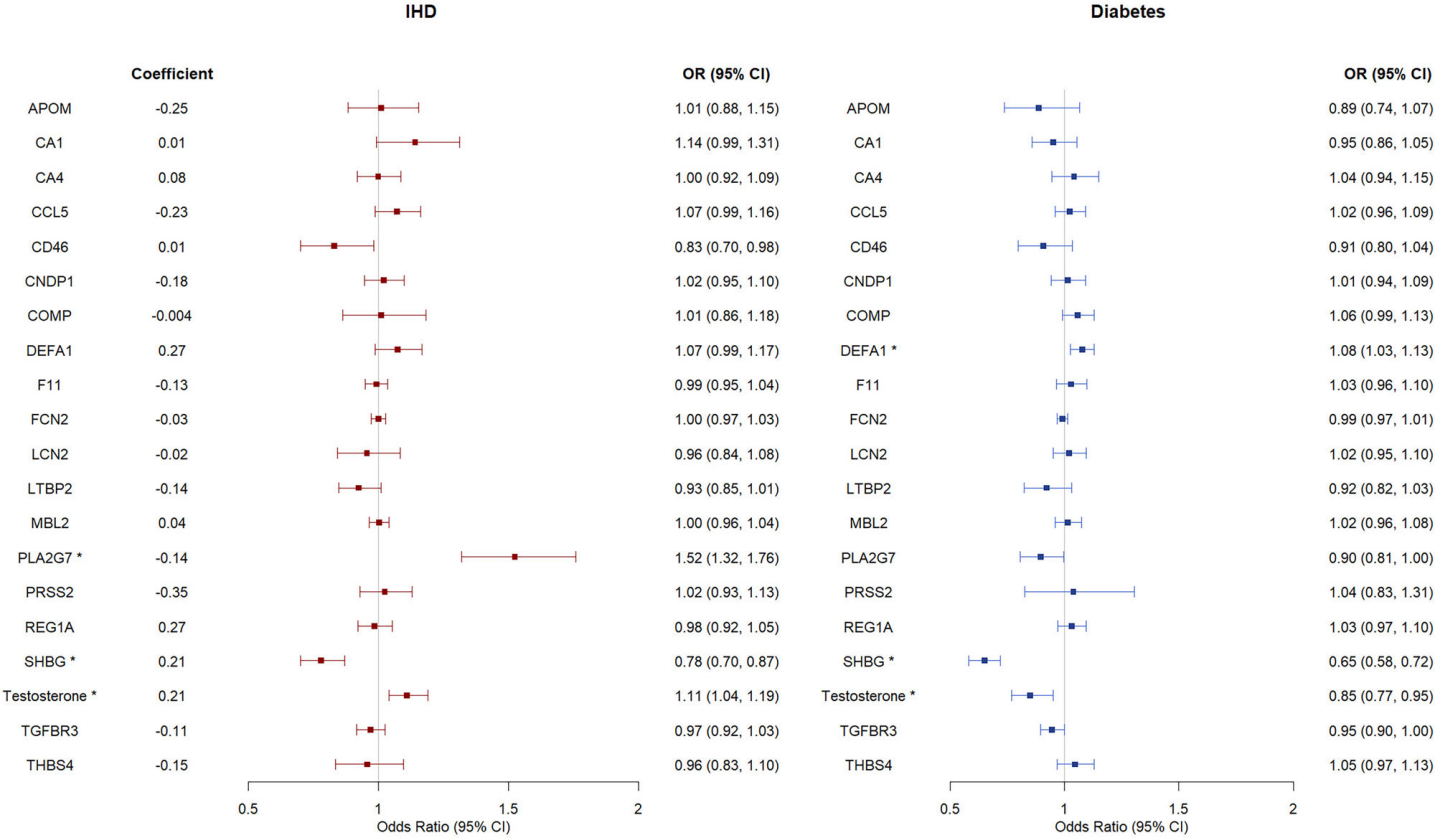
Mendelian randomization analyses on the associations of each protein/sex hormone in berberine signature with IHD and diabetes in men in UK Biobank (for IHD cases = 31,127) and DIAGRAM (for diabetes cases = 41,846). Inverse variance weighting (IVW) was used in MR analyses. The symbol (^*^) means that the association was statistically significant after correction for multiple testing at FDR < 0.05.

Based on the GWAS for each of the 18 proteins in men we conducted, we identified 4–34 genetic instruments for the proteins (Supplementary Table 5). Considering the consistency between study designs and patterns of association as stated in our selection criteria, we found that PRSS2 and SHBG may explain the beneficial role of berberine in IHD (Figs. 4 and 5 and Supplementary Table 6). SHBG showed statistical significance after multiple testing in both logistic regression and MR. For PRSS2, MR showed consistent directions of associations as logistic regression, but the confidence intervals included the null. The association was also inconsistent when using cis-SNPs as instrument, possibly due to the limited number of cis-SNPs (Supplementary Table 7). The associations were similar when using different MR methods and using all SNPs (Supplementary Fig. 7). DEFA1 was positively associated with berberine and associated with higher risk of IHD, TGFBR3 was negatively associated with berberine and associated with lower risk of IHD (Supplementary Table 6); these inconsistent patterns do not align with the overall beneficial association of berberine signature with IHD.

Regarding diabetes, CCL5, CNDP1, F11, LCN2, THBS4, SHBG and testosterone may explain the beneficial association of berberine with diabetes. All associations showed statistical significance after multiple testing in logistic regression (Fig. 4). SHBG and testosterone also showed significant associations in MR. For CCL5, CNDP1, F11 and LCN2, MR analyses showed consistent directions of associations but the confidence intervals included the null (Fig. 5). The associations were also in consistent directions when using cis-SNPs as instruments for the proteins (Supplementary Fig. 8), but had wider confidence intervals. The findings for CCL5, CNDP1, F11, LCN2, and THBS4 in MR using IVW included the null, but the associations of F11 with IHD were statistically significant using weighted median, weighted mode, MR PRESSO and GSMR (Supplementary Fig. 9). SHBG and testosterone were associated with diabetes in both study designs (Figs. 4 and 5). DEFA1 and REG1A were positively associated with berberine and associated with higher risk of diabetes, APOM, PLA2G7, and TGFBR3 were lowered by berberine and associated with lower risk of diabetes (Supplementary Table 6), which does not align with the overall beneficial association of berberine signature with diabetes.

## Discussion

To our knowledge, this is the first study to develop a proteomics signature for berberine intake and show that berberine signature is related to lower risk of IHD and diabetes in men, suggesting that berberine is a promising drug for the prevention and treatment of IHD and diabetes. In this study, we also found that SHBG and PRSS2 may contribute to the observed beneficial association with IHD, while testosterone, SHBG, CCL5, CNDP1, F11, LCN2, and THBS4 may contribute to the observed beneficial associations with diabetes, which may be used as potential targets in new drug development or drug repurposing.

Our findings are consistent with previous trials which showed that berberine are beneficial for lowering lipids^5^ and improving glucose metabolism^7^. Despite, to our knowledge, no RCT has been conducted to examine the effect of berberine on IHD and diabetes. Our study, for the first time, suggests that berberine may lower the risk of IHD and diabetes. The MR effect size of berberine on IHD (OR 0.85) is modest. However, it is comparable to the effect sizes observed for ezetimibe (risk ratio 0.88)^17^ and fibrates (OR 0.78)^18^, both of which are considered adjunctive therapies in lipid management. While statins remain the most effective agents (OR 0.69)^19^, berberine’s potential benefit is notable, especially in people with statin intolerance. Moreover, berberine may offer additional glucose-lowering effects not captured by lipid metrics alone. Several biological pathways may exist. First, berberine lowers lipids and glucose, which are known risk factors for IHD and diabetes. Second, berberine may exert the beneficial effect on IHD via the inhibition of PCSK9. Berberine decreases PCSK9 expression in HepG2 cells^20^. PCSK9 inhibitors are known lipid-lowering drugs and have been shown to lower the risk of IHD^21^. However, their effect on diabetes is unclear^22^, so it may not explain its beneficial effect on diabetes.

In our study, we found that berberine may affect the risk of IHD and diabetes via sex hormones and several proteins, including PRSS2 for IHD, and CCL5, CNDP1, F11, LCN2, and THBS4 for diabetes. Specifically, berberine may lower the risk of IHD via increasing SHBG, and lower the risk of diabetes via increasing SHBG and testosterone. Previous MR studies, including ours, have shown that testosterone increases the risk of IHD^23,24^ but lowers the risk of diabetes in men^25^. In contrast, SHBG lowers the risk of both IHD and diabetes in men^25,26^.

Regarding the proteins underlying the beneficial associations with IHD, PRSS2 regulates lipid metabolism by altering the expression of related genes^27^, which may explain its observed associations with IHD in our study. Regarding the proteins underlying the beneficial associations with diabetes, animal experiment supports that berberine suppresses *CCL5* gene expression^28^, which may lead to lower CCL5 level. CCL5 altered secretion of glucagon-like peptide hormones in animal experiment^29^, and was associated with higher risk of type 2 diabetes in case-control studies in humans^30^, but the causality remains to be clarified. Similarly, berberine reduced *LCN2* expression^31^. LCN2 is an inflammatory biomarker closely related to obesity, insulin resistance, hyperglycemia and diabetes^32,33^, although the causality remains to be established. The role of CNDP1 was partly supported by genetic evidence showing an association with diabetic nephropathy^34^. Thrombospondin-4 (THBS4) is a member of thrombospondins and has been identified as a secreted protein regulating cell communications and inflammation, such as TGF-beta^35^; TGF-beta signaling regulates glucose tolerance and energy homeostasis^36^. Berberine may lower THBS4 by inactivating the SMAD3 pathway^37^, which is involved in the regulation of THBS4 levels^38^. Berberine also has activity in inhibiting thrombin-induced platelet aggregation^39^, and lowers F11^40^, a serine protease involved in the intrinsic coagulation cascade. Coagulation factors may be involved in diabetes, and F11 level has been shown to be elevated in patients with diabetes^41^.

Integrating randomized controlled trial, cohort study and MR, our novel study provided timely evidence regarding the role of berberine on IHD and diabetes. We also conducted analyses on lipid fractions, which provided a deeper and more comprehensive understanding on the role of berberine on lipids. Despite, we acknowledge that we have several limitations. First, the berberine signature was derived from a relatively small, short-term RCT conducted in East Asian. While the RCT design provides a strong basis for inferring causality for the proteomic changes, the limited sample size may have limited the statistical power to detect smaller protein alterations, and the short intervention period does not capture potential long-term or adaptive biological responses, although the duration is used in most berberine trials^7^. Second, the AUC for the elastic net mode is modest, which may raise concerns of potential overfitting. However, elastic net can mitigate overfitting by balancing feature selection and coefficient shrinkage^42^. To further assess robustness, we conducted biological validation by comparing associations of the signature with established clinical biomarkers against evidence from RCTs. The signature shows favorable associations with blood lipids and glucose (Table 1), consistent with the known effects of berberine reported in RCTs^43,44^, none of which were included in model training. This supports that the model captures biologically relevant signals rather than noise. The robustness was also supported by the permutation analysis and the consistency across folds. Replication in an independent cohort would further strengthen confidence. While no comparable berberine trial with proteomic data is currently available, our findings provide a unique resource for future validation studies. Third, we applied this signature to an independent cohort that was predominantly of European ancestry. Differences in genetic background, lifestyle, environmental exposures, baseline proteomics and technical assessment batches between these populations could affect the generalizability of the signature and lowering the precision of the estimates. However, the directions of associations based on RCT are expected to be consistent across settings, for example, the effects of berberine on total cholesterol are similar in trials conducted in Italy (−0.16 mmol/L, 95% confidence interval (CI) −0.25, −0.06)^45^ and in mainland China (−0.16 mmol/L, 95% CI)^46^, with overlapping confidence intervals. It would be worthwhile to replicate the analyses in cohorts of the same ancestry. While there are clinical trials investigating berberine in people of European ancestry (e.g., Fogacci et al.^47^, Fogacci et al.^48^, Derosa et al.^45^), some studies used berberine in combination with other supplements rather than as a standalone intervention^47,48^, and none provided proteomic data similar to ours. Furthermore, we were unable to identify any large-scale, publicly available biobanks of East Asian populations with individual-level data and proteomic measurements similar to those available in the UK Biobank. These constraints limit our ability to validate the findings across ancestries. Given these limitations, our results should be interpreted as exploratory rather than definitive. They provide preliminary evidence suggesting potential cardiometabolic benefits of berberine and highlight the need for future, adequately powered trials to confirm these associations and establish causality, given the current absence of such trials. Until such data become available, our findings should be viewed as hypothesis-generating and as a rationale for further trial rather than as conclusive evidence. Fourth, our findings are based on men and may not be directly generalizable to women. Evidence from our previous meta-analyses of randomized controlled trials suggests that berberine has a larger effect on glucose metabolism biomarkers in women than in men^7^, while its effects on lipid profiles appear similar across sexes^5^. Applying the same analysis to women using existing data (see Supplementary Table 8) could therefore underestimate the true effect size in women. A trial in women would provide more accurate estimates. We are currently conducting a clinical trial in women in Hong Kong and seeking funding to perform the same proteomic assays. This will enable us to generate more precise estimates in women using similar analytical methods once these data become available. Although our current study cannot precisely model sex-specific associations, given the stronger metabolic benefits observed in women^7^, it is plausible that berberine would also be associated with a lower risk of IHD and diabetes in women. Fifth, MR can minimize confounding, but the estimates are less precise than conventional observational study, as the genetic variants only account for a small proportion of the variance in the exposure^49^. Considering the limited number of genetic instruments, the inconsistencies between observational and MR analyses, especially in significance, we should interpret the MR results with caution. Replication in a larger MR is worthwhile. Sixth, our analysis focused on identifying proteins that respond to berberine and show consistent associations with cardiometabolic disease risk. We did not conduct a formal statistical analysis involving all three variables (berberine, protein levels, and disease risk) in one dataset because these associations were derived from distinct datasets and study designs. Specifically, berberine-protein associations were assessed using clinical trial data and elastic net regression, while protein-disease associations were obtained through observational and Mendelian randomization analyses in the UK Biobank. This integrative approach identified potential mediating proteins but does not quantify the proportion of mediation. A large, adequately powered randomized controlled trial of berberine incorporating measurements of these proteins and clinical outcomes would be necessary to address this question. Seventh, it is possible that other proteins may underlie the effect of berberine on IHD and diabetes. As such, including more proteins on cardiometabolic diseases is worthwhile in future studies. Finally, the findings on lipid fractions should be interpreted with caution. The observed associations of berberine signature with lower particle concentrations of small and medium HDL may reflect complex metabolic associations rather than straightforward health effects. Currently the causa role of HDL in IHD is also uncertain^50^, it seems that HDL diameter and cholesterol levels in very large HDL were associated with IHD^51^, whilst we did not observe associations with these HDL fractions in our study.

From the perspective of clinical practice, berberine has got arising attention as lipid-lowering treatment. This study highlights the need for large randomized controlled trials to evaluate the cardiometabolic effects of berberine and confirm the associations observed here, which could have important implications for clinical practice. From the perspective of public health, berberine is commonly used as a nutrient supplement in the US. So, for the benefits of the general public, it is helpful to clarify its role in IHD and diabetes. We answered this question using multiple study designs. We also identified several proteins and sex hormones accounting for these observed associations, which can be used as modifiable targets in new drug development or drug repurposing.

## Methods

### Study design

In this study, leveraging the proteomics and sex hormones data in the completed randomized controlled trial of berberine in men (Fig. 1), we built a signature reflecting the response to berberine use (in short “berberine signature”). The trial was a parallel, placebo-controlled trial in 80 men, aiming to examine the effect of berberine on cardiovascular disease risk factors, with details in the previous publication^11^. As our previous trial showed that berberine lowered total cholesterol, fasting glucose and possibly LDL-cholesterol in men, for verifying the signature effect, we examined the association of the berberine signature with lipids and glucose in the trial and also in men in UK Biobank. We hypothesized that the berberine signature had an inverse association with total cholesterol, LDL-cholesterol and fasting glucose.

We then assessed whether this signature was associated with the risk of IHD and diabetes in UKB proteomics study. We also examined the causal relationship using MR in UK Biobank and DIAbetes Genetics Replication And Meta-analysis (DIAGRAM). MR uses genetic variants as instrument to minimize confounding. As genetic variants are randomly allocated at conception, they are not affected by socioeconomic positions or lifestyle, therefore can provide an estimate for causal inference^16^. To conduct MR analysis, we conducted a genome-wide association study (GWAS) of the berberine signature, and examined whether genetically predicted berberine signature was associated with IHD and diabetes in men of UK Biobank and DIAGRAM respectively. We also examined the reverse direction of association, i.e., the association of IHD and diabetes with berberine signature. To further examine the role of berberine in lipid metabolism, we also examined the association of berberine signature with lipid fractions in men of UK Biobank, measured using nuclear magnetic resonance (NMR). We conducted both linear regression and MR analyses in UK Biobank.

To further identify the specific protein(s) or sex hormone(s) accounting for the associations with IHD and diabetes, we further examined the associations of each protein and sex hormone with IHD and diabetes using logistic regression and MR. When conducting MR analysis, we first conducted GWAS of each protein included the berberine signature in men in UK Biobank, to obtain genetic instruments for each protein in men, then we examined the association of genetically predicted protein with IHD and diabetes^52^. For the role of sex hormones in IHD and diabetes in MR, we referred to the previous publications by us^23,26^ and by others^25^. The flow chart of the study was shown in Fig. 1.

### Ethics approval

The clinical trial of berberine has obtained ethical approval from the Institutional Review Board of the University of Hong Kong/Hospital Authority Hong Kong West Cluster (UW18-037). All participants provided informed written consent. The data from UK Biobank was obtained from the UK Biobank Resource under application numbers 42,468 and 45,052. The UK Biobank has received the ethical approval from North West Multi-centre Research Ethics Committee (MREC), which covers the UK. It also got the approval from the Patient Information Advisory Group (PIAG) in England and Wales, and from the Community Health Index Advisory Group (CHIAG) in Scotland. Informed written consent was given prior to the inclusion of subjects in the study. The study conforms to the ethical guidelines of the 1975 Declaration of Helsinki. The analysis of other publicly available summary statistics does not require additional ethical approval.

### Building berberine signature based on our completed berberine trial in men

In our completed RCT (registration number NCT03770325, registered in ClinicalTrials.gov on Dec 2018), we included 84 Chinese men with hyperlipidemia in Hong Kong (sample size calculation shown in our published study^11^), according to the following criteria:

1. aged 20–65 years;
2. of Chinese ethnicity;
3. with hyperlipidemia with TG greater than 150 mg/dl (1.70 mmol/L), total cholesterol greater than 200 mg/dl (5.16 mmol/L), and/or LDL-cholesterol greater than 100 mg/dl (2.58 mmol/L)^53^;
4. willing to make return visits;
5. not currently receiving hormone replacement therapy, such as testosterone replacement therapy, in the past 12 months;
6. not currently taking berberine or nutraceuticals that contain berberine;
7. free of any congenital diseases, including familial hypercholesterolemia;
8. free of any infectious diseases, e.g., seasonal influenza;
9. without liver/renal dysfunction.

For the participants satisfying the inclusion criteria, after obtaining their informed consent, each man was assigned a study number and randomly allocated to the berberine group or placebo group. The allocation was according to a computer-generated random number, prepared by a statistician. All other investigators and all participants were blinded to the allocation to control or intervention at 1:1 ratio. At the baseline assessment, the participant filled out a questionnaire including medical history, smoking status, alcohol use, physical activity, and socio-demographics, and was provided with the package of drugs prepared by the statistician, containing berberine or placebo. A small amount of blood (~20 mL) was drawn into several vacutainers. The men took berberine (500 mg orally twice a day) or placebo tablets, prepared with the same appearance, for 12 weeks. At 8 and 12 weeks after intervention, the participants returned for follow-up, including blood taking.

All 84 men (mean age 46.9 years old) attended the baseline assessment, 80 men attended the follow-up after 8 and 12 weeks. In total, 4 men (2 in the berberine group and 2 in the placebo group) dropped out due to lack of time or for personal reasons. The baseline characteristics were shown in our published study^11^. Blood samples were used for the assessment of lipid profile and sex hormones. Using generalized estimating equations (GEE) model, the analysis showed berberine may reduce total cholesterol, LDL-cholesterol, and increase sex hormone binding globulin and testosterone^11^. The rest of the blood samples have been stored at −80 °C and then were used to measure proteomics and lipid fractions. Proteomics of 92 proteins in cardiometabolic panel were measured using OLINK platform. Lipid fractions were measured using Nightingale Metabolomics platform. The same platforms have been used in large biobanks such as UK Biobank.

When building the berberine signature, we first standardized all proteins and sex hormones to the same scale and then used elastic net^54^ to regress the use of berberine on the 92 proteins and two sex hormones (testosterone and SHBG), to build the berberine signature. We also included testosterone and SHBG as we found berberine affected sex hormones in our previous analyses^11^ and previous studies^14,15^. The berberine signature was calculated as the weighted sum of the selected proteins and sex hormones, with weights equal to coefficients from the elastic net regression. To avoid overfitting, we used a leave-one-out cross-validation approach, with a predetermined alpha parameter of 0.5. To evaluate the robustness of the selected protein associations, we performed a permutation analysis by randomly shuffling berberine treatment labels and refitting the elastic net model 500 times. It generated a reference distribution of coefficients expected under no true association. For each selected protein, we calculated an empirical p-value by comparing the observed coefficient to the distribution obtained from the permuted models, defined as the proportion of permutations in which the absolute coefficient exceeded that of the observed model. We additionally calculated, for each protein or sex hormone, its selection frequency across the 80 cross-validation folds, the corresponding percentage, and the number of times its coefficient sign was consistent with that in the signature.

### Conduct GWAS of berberine signature

UK Biobank is a large, ongoing, prospective cohort study, with currently a median follow-up time of 11.1 years^55^. It recruited 502,713 participants (aged 40–69 years, mean age 56.5 years, 45.6% men) from 2006 to 2010 in England, Scotland, and Wales, with 94% of self-reported European ancestry. UK Biobank Pharma Proteomics Project (UKB-PPP) is a precompetitive consortium of 13 biopharmaceutical companies that funded the generation of multiplex proteomic data in 54,219 UKB participants. The details of UKB-PPP were shown in the previous publication^56^. A total of 2923 unique proteins across eight protein panels (cardiometabolic, cardiometabolic II, inflammation, inflammation II, neurology, neurology II, oncology, and oncology II in the antibody-based Olink Explore 3072 platform were measured, with quality control^56^.

We calculated the berberine signature using the formula built in the previous step, and performed a GWAS of the berberine signature in the UK Biobank using REGENIE^57^.

For quality control, the imputed genomic data were filtered using the following criteria: (1) the excluding variants with an imputation quality (INFO) score below 0.7; (2) excluding those with missing genotype rates exceeding 10%; (3) excluding those with minor allele frequency (MAF) less than 0.01; and (4) excluding Hardy-Weinberg Equilibrium (HWE) *p*-value below 1e-15. REGENIE was utilized to perform GWAS analyses through a two-step approach. Initially, a whole-genome regression model predicted individual traits using the leave-one-chromosome-out (LOCO) method and genotyped variants meeting criteria, complemented by linkage disequilibrium pruning (1000 variant windows, 100 sliding windows, and *r*^2^ < 0.8). In the subsequent variant association analysis, LOCO phenotypic predictions were utilized as offsets, and only quality-controlled variants were used. Berberine levels were inverse rank normalized and used as the phenotype. As previously^56^, the association models incorporated several covariates, including age, the square of age (age^2^) to capture non-linear effects, batch effects, the UK Biobank assessment center, the type of genetic array used, and the first 20 principal components to adjust for population stratification.

### Assess the association of berberine signature with lipids and glucose in the trial and in UK Biobank using linear regression and MR

We examined the association of berberine signature with changes in total cholesterol, fasting glucose, and LDL-cholesterol in the berberine trial, using linear regression. We also examined the associations of berberine signature with total cholesterol, fasting glucose, and LDL-cholesterol in men in UK Biobank, controlling for age, ethnicity, smoking, Townsend index, education, and physical activity. To investigate potential causality, we also used MR analysis to assess the association of genetically predicted berberine signature with these biomarkers in men.

We obtained genetic association with total cholesterol, fasting glucose, and LDL-cholesterol in GWAS summary statistics in men in UK Biobank, provided by Neale Lab (https://www.nealelab.is/uk-biobank), and the association with berberine signature from the GWAS we conducted. In the MR analysis, we meta-analyzed the Wald estimate for each SNP using inverse variance weighting (IVW). We also used other analytic methods more robust to pleiotropy, including weighted median^58^, weighted mode^59^, MR-Egger^60^, Mendelian Randomization Pleiotropy RESidual Sum and Outlier (MR-PRESSO)^61^, and Generalised Summary-data-based Mendelian Randomisaion (GSMR)^62^. The weighted median provides a consistent estimate of the causal effect even when up to 50% of the information is from genetic variants that are invalid instruments^58^. The weighted mode is based on the assumption that a plurality of genetic variants are valid instruments, i.e., no larger subset of invalid instruments estimating the same causal estimate than the subset of valid instruments exists^59^. MR-Egger can assess whether genetic variants have pleiotropic effects on the outcome that differ on average from zero (directional pleiotropy), and provide a consistent estimate of the causal effect^60^. MR-PRESSO and GSMR both tried to identify the genetic variant(s) differentially driving the associations, i.e., outliers^61,62^, and provide corrected estimates removing the outliers.

### The role of berberine signature in IHD, diabetes, and lipid fractions in UKB proteomics study and MR

To examine the association of berberine signature with IHD and diabetes, we used logistic regression with adjustment for age, ethnicity, smoking, Townsend index, education, and physical activity in the UK Biobank.

For the MR analyses, we used both one-sample and two-sample MR methods. Using one-sample MR method, we used logistic regression to examine the association of the genetic score with IHD and diabetes in UK Biobank, where the genetic score was comprised of the six genetic instruments after removing the potentially pleiotropic SNPs (details shown in Supplementary Table 2). We also obtained the association of each SNP in the genetic score with IHD in men in UK Biobank, and with diabetes in a published GWAS of diabetes in men in DIAGRAM^52^, and used different analytic methods as above, including IVW, weighted median, weighted mode, MR-Egger, MR-PRESSO, and GSMR. In UK Biobank, we identified the cases of IHD from record linkage to hospitalization and death records, as well as from a nurse-led interview at recruitment, as previously^63^. The genetic associations with diabetes in men were obtained from summary statistics of a GWAS meta-analysis from DIAGRAM, including 41,846 cases and 383,767 controls^52^. The participants from DIAGRAM did not overlap with UK Biobank.

In the bi-directional MR analysis on berberine signature and IHD and diabetes, we also examined the other direction, i.e., the association of IHD and diabetes with berberine signature. Here, we obtained genetic instruments for IHD and diabetes from published GWAS in CARDIoGRAMplusC4D and DIAGRAM^52,64^, respectively. We selected genetic variants with genome-wide significance and clumped at *r*^2^ of 0.001. In the MR analyses, we used IVW and other sensitivity analyses methods, including weighted median, weighted mode, MR Egger, MR-PRESSO, and GSMR.

To have deeper understanding of the effect of berberine on lipids, we also examined the effect of berberine on lipid fractions using GEE model (*n* = 80), as well as the associations of berberine signature with lipid fractions in the berberine trial (*n* = 80) and in men in UK Biobank (*n* = 3750). The lipid fractions were measured using NMR spectroscopy in Nightingale platform^65^. We also used MR analyses in men of UK Biobank (*n* = 90,558) to assess whether the associations are causal. The lipid fractions included the concentrations of various subclasses of HDL, LDL, intermediate density lipoprotein (IDL), and very-low density lipoprotein (VLDL) particles, and cholesterol and triglyceride content of these particles. To avoid sample overlap, we excluded participants who had berberine signature data in the MR analyses. We identified lipid fractions with statistical significance in UK Biobank linear regression and MR analyses after correction for multiple testing (FDR < 0.05) and with consistent directions in the berberine trial.

### The role of individual proteins and sex hormones in IHD and diabetes

To identify the protein(s) and sex hormone(s) accounting for the associations of berberine signature with IHD and diabetes, we examined the association of each protein and sex hormone in the berberine signature with IHD and diabetes, to select protein(s) and sex hormone(s) that (1) negatively correlated with berberine signature, and associated with higher risk of IHD and/or diabetes; (2) positively correlated with berberine signature, and associated with lower risk of IHD and/or diabetes and (3) the association with IHD and/or diabetes in logistic regression reached statistical significance after multiple testing (FDR < 0.05) and showed consistent directions of associations in MR. Proteins showing inconsistent directions of associations, such as those positively correlated with the berberine signature but also with higher risk of IHD and/or diabetes, were excluded from potential mediators. These patterns may represent off-target effects of berberine rather than mechanisms underlying its beneficial associations with IHD and diabetes.

To examine the role of each protein in IHD and diabetes, first we conducted multivariable logistic regression to assess the association of each protein and sex hormone with IHD and diabetes in UK Biobank, controlling for age, ethnicity, smoking, alcohol drinking, Townsend index, education, and physical activity. Second, we conducted GWAS for each protein in men in UK Biobank and conducted MR analyses on the effect of each protein on IHD and diabetes in men. In the GWAS of each protein, we followed similar procedures and quality control as in the GWAS of berberine signature. Based on the GWAS, we identified genetic instruments for each protein. Specifically, we kept the SNPs reaching genome-wide significance and clumped at *r*^2^ of 0.01, and conducted MR analyses using similar approach as above. In sensitivity analyses, we used only cis-SNPs, i.e., the SNPs in the gene region coding the proteins; specifically, we used SNPs within the gene region or ±100 kb of the gene. The Wald ratio was calculated using genetic association with IHD or diabetes divided by the genetic association with each protein. The role of sex hormones (testosterone and SHBG) in IHD and diabetes in men has been conducted in previous MR studies^23,25,26^, so we cited the estimates from published studies. We only selected proteins associated with IHD or diabetes in logistic regression with false discovery rate (FDR) < 0.05, and with consistent directions of associations in MR analyses.

## Supplementary information

Supplementary Information

## Data Availability

This research has been conducted using the UK Biobank Resource under Application Numbers 42,468 and 45,052 and using the clinical trial data collected by the team of the corresponding author. The UK Biobank data will be available upon request and approval by the UK Biobank (https://www.ukbiobank.ac.uk/enable-your-research/apply-for-access). The request for the clinical trial data should be made by contacting the corresponding author.

## Code availability

The underlying code for this study is not publicly available but may be made available to qualified researchers on reasonable request from the corresponding author. All statistical analyses were conducted using REGENIE, and the “glmnet”, “MRPRESSO”, “gsmr”, and “MendelianRandomization” packages in R (version 4.0.1, R Foundation for Statistical Computing, Vienna, Austria).
