# Supplementary material for: Berberine signature and cardiometabolic diseases using randomized controlled trial, cohort study and Mendelian randomization
Source: NPJ Cardiovasc Health. 2026 Mar 25;3:15. doi: 10.1038/s44325-026-00113-w (PMC13018313; doi:10.1038/s44325-026-00113-w)
Supplement: Supplementary file 1 — Supplementary Information [file 44325_2026_113_MOESM1_ESM.pdf]

Supplemental Figure 1. The receiver operating characteristic (ROC) curve of the berberine signature based on leave-one-out cross-validation

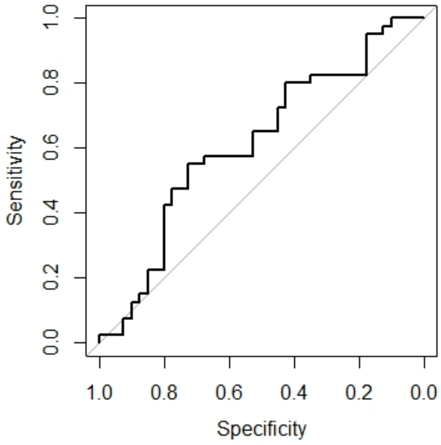

Supplemental Figure 2. Permutation analysis of elastic net coefficients for selected proteins. Histograms show the distribution of coefficients obtained from 500 permutation runs (randomly shuffled berberine treatment labels). Red vertical lines indicate observed coefficients from the original model. Empirical p-values for each protein were also presented.

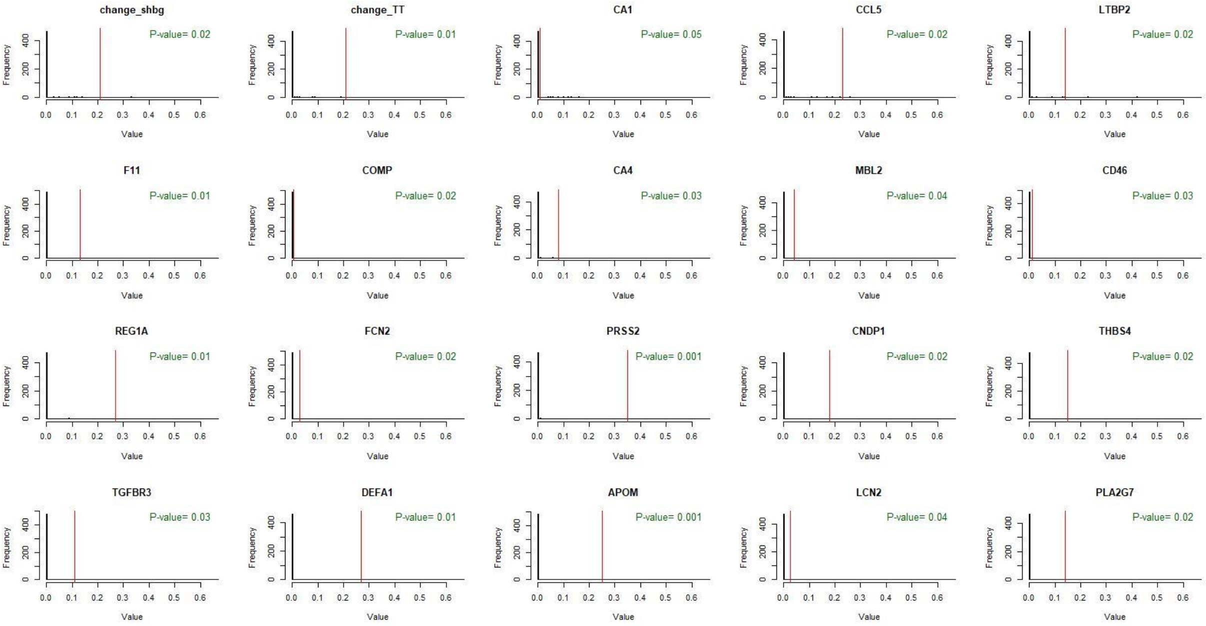

Supplemental Figure 3. Manhattan Plot of the genome-wide association study of berberine signature in 19,413 men

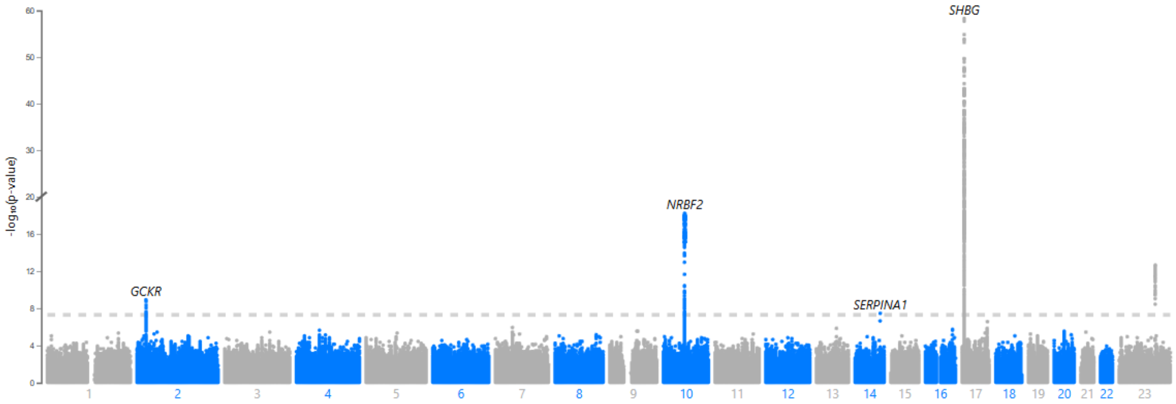

Supplemental Figure 4. Scatter plot on berberine genetic instruments and IHD and diabetes using MR

(a) IHD

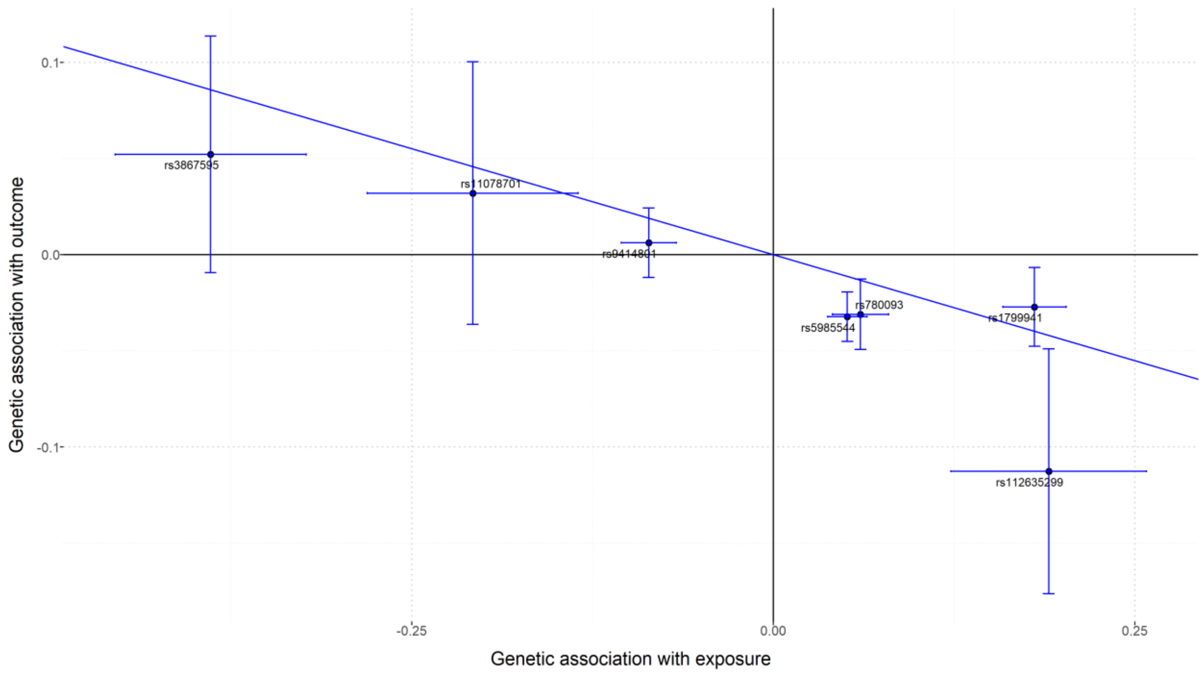

(b) Diabetes

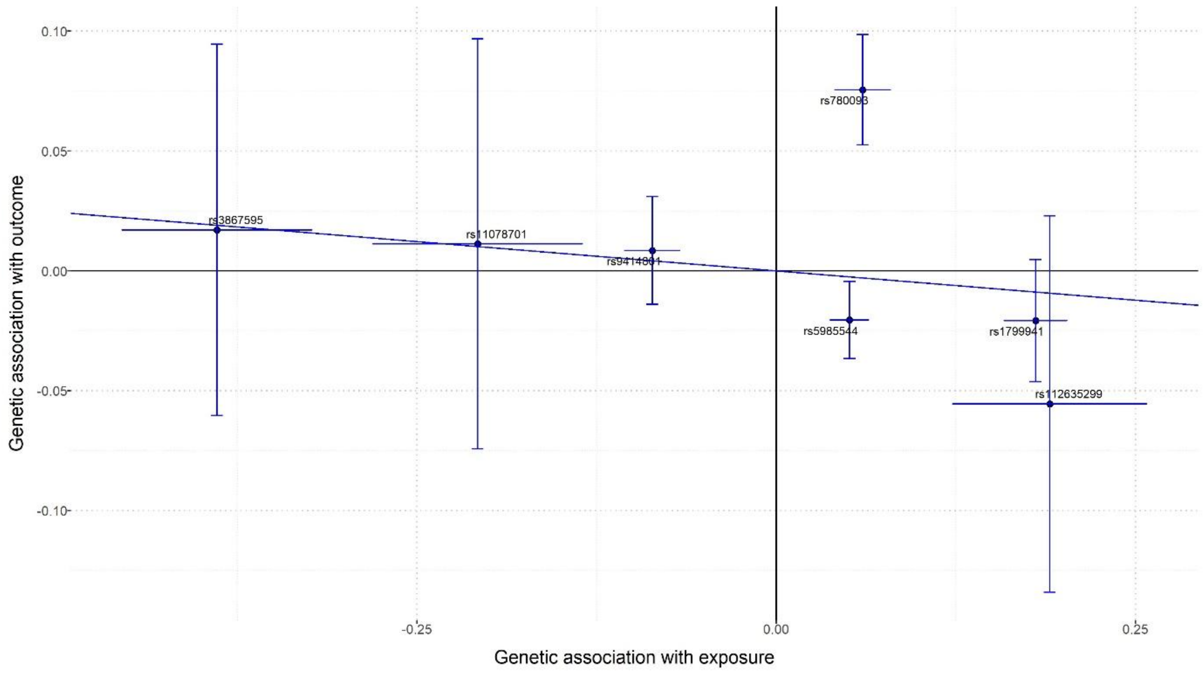

Supplemental Figure 5. The association of genetically predicted berberine signature with lipid fractions using different MR methods in 90,558 men in UK Biobank. Different methods include IVW, weighted mode, MR-Egger, MR-PRESSO, and GSMR.

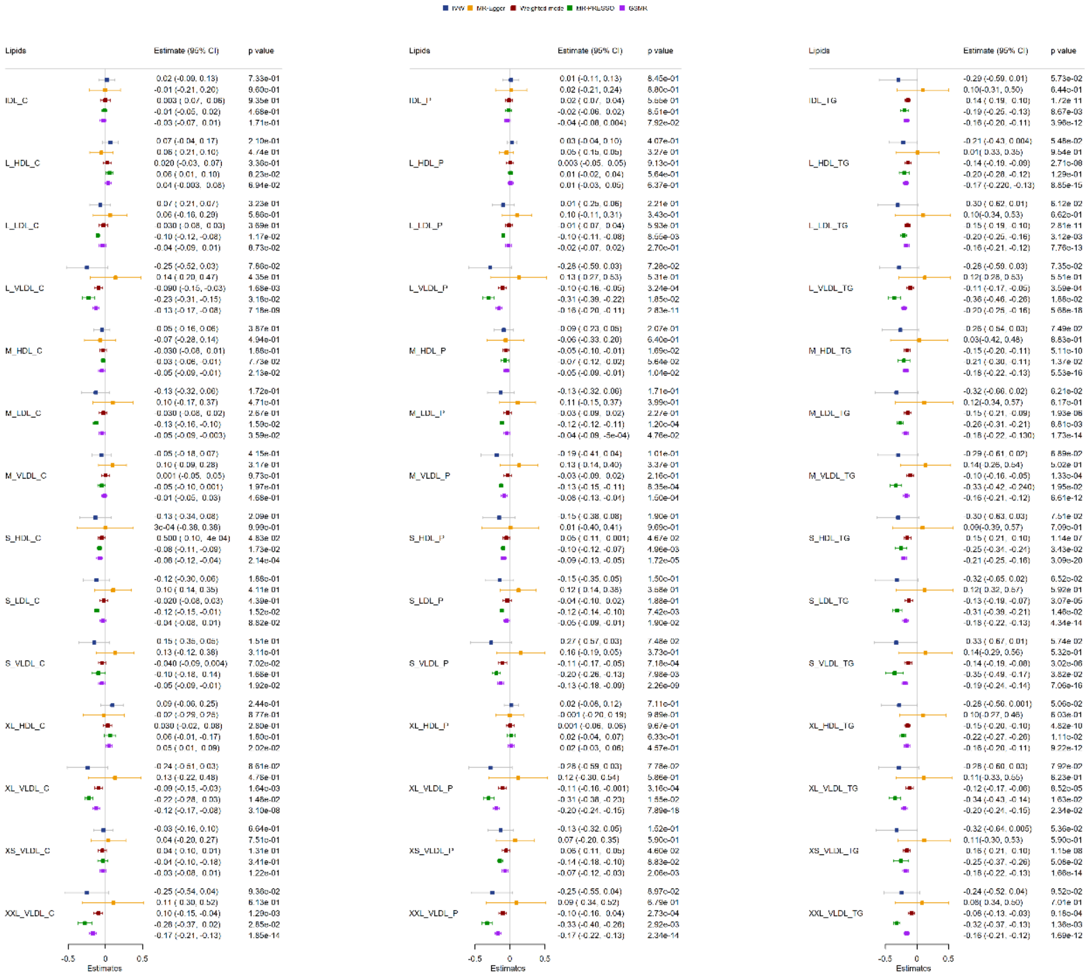

Supplemental Figure 6. The association of individual protein with ischemic heart disease using cis-SNPs as instrument in MR analysis

Protein	Log OR (95% CI) for IHD	p value
APOM	0.003 (-0.07, 0.07)	9.44e-01
CA1	0.17 (-0.06, 0.41)	1.52e-01
CA4	0.01 (-0.05, 0.07)	7.29e-01
CCL5	0.14 (0.03, 0.25)	1.46e-02
CD46	-0.15 (-0.36, 0.05)	1.48e-01
CNDP1	-0.04 (-0.10, 0.01)	1.24e-01
COMP	0.06 (-0.03, 0.14)	1.86e-01
F11	-0.01 (-0.06, 0.04)	7.48e-01
FCN2	-0.01 (-0.04, 0.02)	6.36e-01
LCN2	0.15 (-0.10, 0.41)	2.47e-01
LTBP2	-0.08 (-0.21, 0.06)	2.49e-01
MBL2	0.003 (-0.02, 0.03)	7.96e-01
PLA2G7	-0.17 (-0.44, 0.10)	2.08e-01
PRSS2	-0.16 (-0.29, -0.03)	1.38e-02
REG1A	-0.01 (-0.07, 0.05)	8.23e-01
TGFBR3	-0.06 (-0.17, 0.06)	3.25e-01
THBS4	0.06 (-0.02, 0.14)	1.68e-01

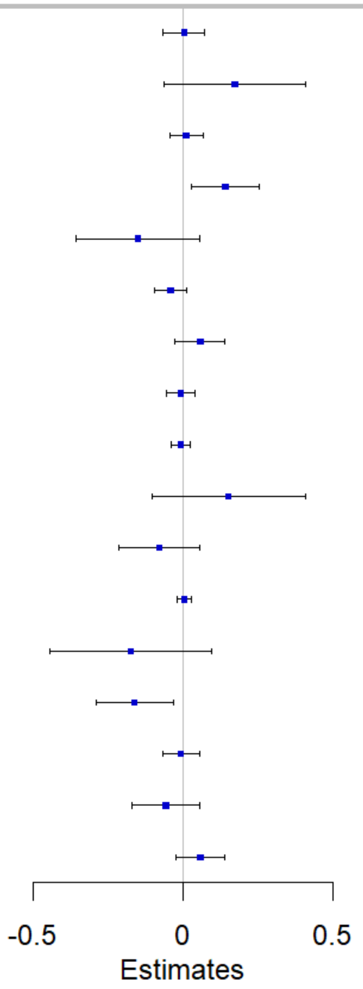

Supplemental Figure 7. The association of individual protein with ischemic heart disease using different MR methods based on all available SNPs as instrument. Different methods include IVW, weighted median, weighted mode, MR-Egger, MR-PRESSO, and GSMR

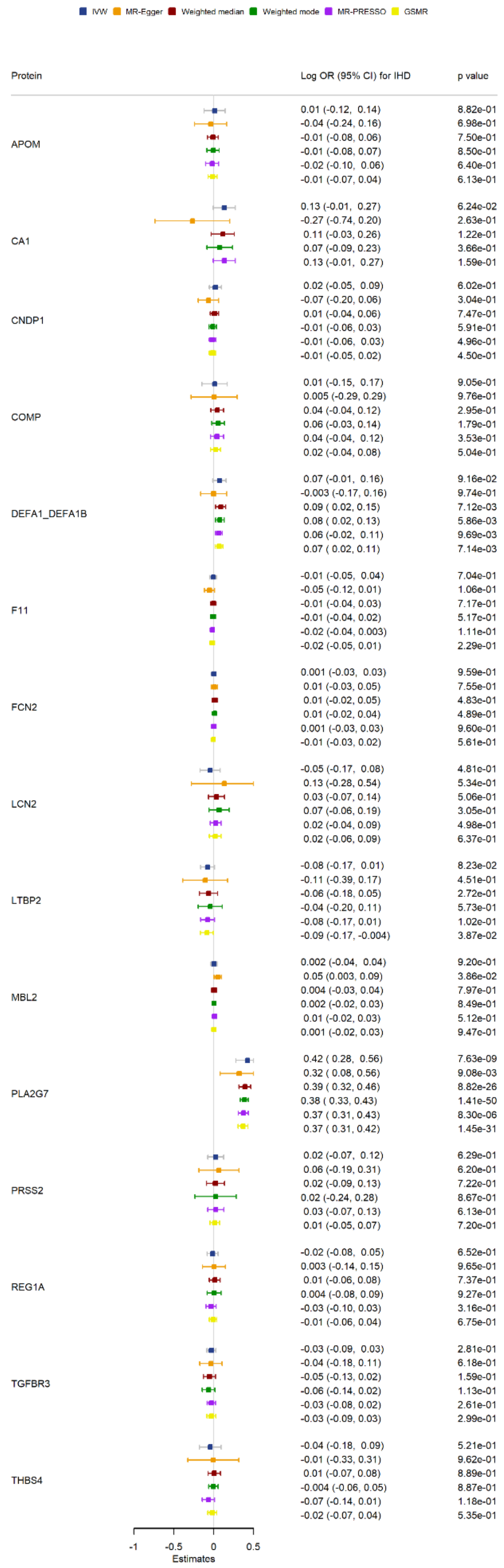

Supplemental Figure 8. The association of individual protein with diabetes using cis-SNPs as instrument in MR analysis

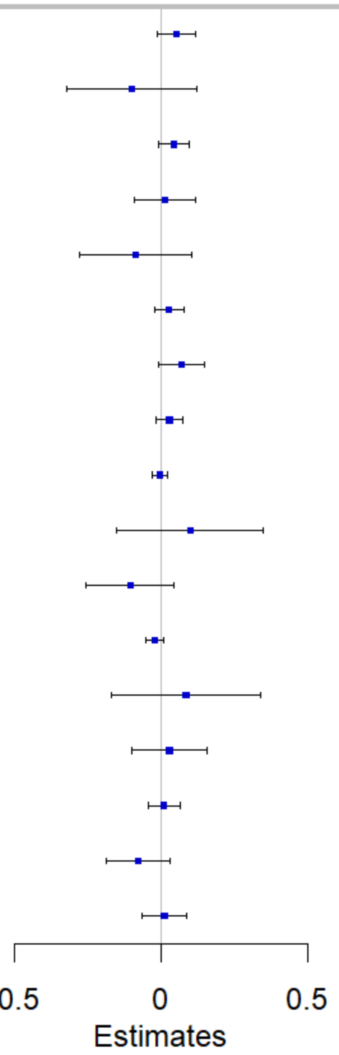

Protein	Log OR (95% CI) for Diabetes	p value
APOM	0.05 (-0.01, 0.12)	1.11e-01
CA1	-0.10 (-0.32, 0.12)	3.71e-01
CA4	0.04 (-0.01, 0.09)	1.00e-01
CCL5	0.01 (-0.09, 0.12)	8.20e-01
CD46	-0.09 (-0.28, 0.10)	3.71e-01
CNDP1	0.03 (-0.02, 0.08)	2.87e-01
COMP	0.07 (-0.01, 0.15)	9.17e-02
F11	0.03 (-0.02, 0.07)	2.22e-01
FCN2	-0.003 (-0.03, 0.02)	8.03e-01
LCN2	0.10 (-0.15, 0.35)	4.40e-01
LTBP2	-0.11 (-0.26, 0.04)	1.69e-01
MBL2	-0.02 (-0.05, 0.01)	1.30e-01
PLA2G7	0.08 (-0.17, 0.34)	5.16e-01
PRSS2	0.03 (-0.10, 0.15)	6.65e-01
REG1A	0.01 (-0.04, 0.06)	7.45e-01
TGFBR3	-0.08 (-0.19, 0.03)	1.51e-01
THBS4	0.01 (-0.07, 0.09)	7.87e-01

Supplemental Figure 9. The association of individual protein with diabetes using different MR methods based on all available SNPs as instrument. Different methods include IVW, weighted median, weighted mode, MR-Egger, MR-PRESSO, and GSMR

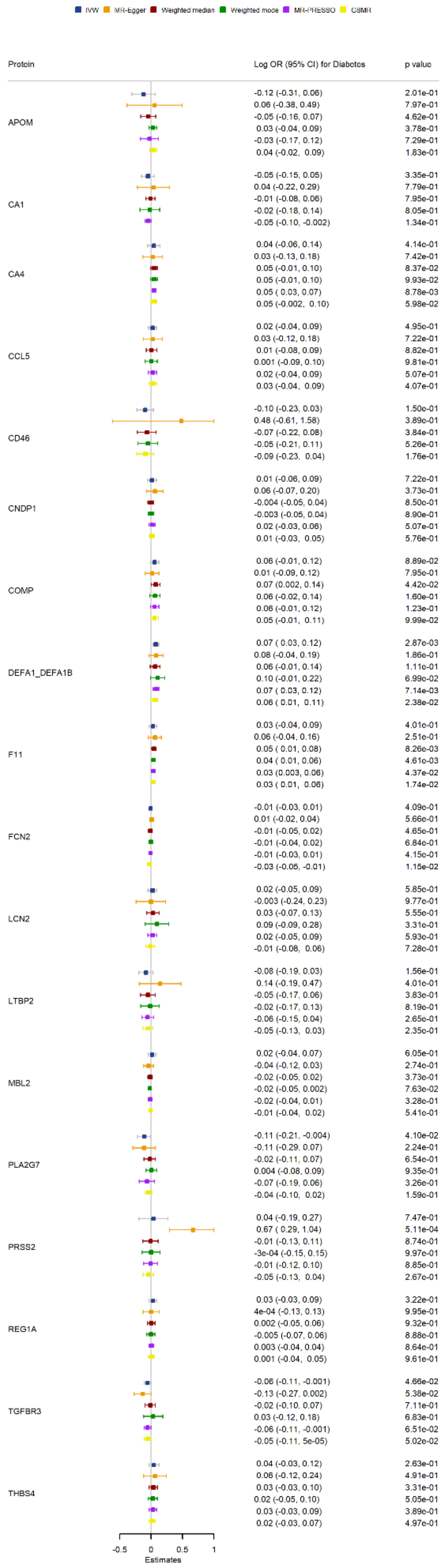

Supplemental Table 1. Selection frequency and sign consistency of selected proteins and sex hormones

Selected proteins/sex hormones	Frequency	percentage	Sign consistency	Consistency percentage
APOM	79	98.8%	79	100%
CA1	34	42.5%	34	100%
CA4	70	87.5%	70	100%
CCL5	79	98.8%	79	100%
CD46	49	61.3%	49	100%
SHBG	79	98.8%	79	100%
Testosterone	79	98.8%	79	100%
CNDP1	79	98.8%	79	100%
COMP	45	56.3%	45	100%
DEFA1	79	98.8%	79	100%
F11	79	98.8%	79	100%
FCN2	67	83.8%	67	100%
LCN2	52	65.0%	52	100%
LTBP2	79	98.8%	79	100%
MBL2	52	65.0%	52	100%
PLA2G7	79	98.8%	79	100%
PRSS2	79	98.8%	79	100%
REG1A	78	97.5%	78	100%
TGFBR3	78	97.5%	78	100%
THBS4	79	98.8%	79	100%

Supplemental Table 2. Genetic instruments for berberine signature

rsid	effect_allele	chromosome	beta	se	pval	note
rs11078701	T	17	-0.21	0.04	2.28E-08	
rs112635299	T	14	0.19	0.03	3.35E-08	
rs1799941	A	17	0.18	0.01	5.57E-59	
rs3867595	C	17	-0.39	0.03	7.25E-31	
rs5985544	T	23	0.05	0.01	2.19E-13	excluded in the analyses for IHD
rs780093	C	2	0.06	0.01	1.23E-09	excluded in the analyses for diabetes
rs9414801	A	10	-0.09	0.01	8.79E-19	

Supplemental Table 3. Genetic instruments for ischemic heart disease in the bi-directional MR analysis

SNP	effect_allele	chromosome	beta	se	pval
rs10080815	G	6	0.25	0.03	1.33E-15
rs10139550	G	14	0.06	0.01	1.38E-08
rs10840293	A	11	0.05	0.01	1.28E-08
rs11065979	T	12	0.07	0.01	1.93E-10
rs11191416	G	10	-0.08	0.01	4.65E-09
rs11556924	T	7	-0.07	0.01	5.34E-11
rs115654617	A	2	0.14	0.02	3.12E-18
rs11838776	A	13	0.07	0.01	1.83E-10
rs1199338	C	3	0.07	0.01	3.90E-09
rs12202017	G	6	-0.07	0.01	1.98E-11
rs1412444	T	10	0.07	0.01	5.15E-12
rs16986953	A	2	0.09	0.02	1.45E-08
rs17087335	T	4	0.06	0.01	4.59E-08
rs17678683	G	2	0.10	0.02	3.00E-09
rs180803	T	22	-0.18	0.03	1.64E-10
rs1870634	G	10	0.08	0.01	5.55E-15
rs2107595	A	7	0.07	0.01	8.05E-11
rs2128739	C	11	-0.07	0.01	7.05E-11
rs2487928	A	10	0.06	0.01	4.41E-11
rs2519093	T	9	0.08	0.01	1.19E-11
rs2681472	G	12	0.07	0.01	6.17E-11
rs28451064	A	21	0.13	0.02	1.33E-15
rs2891168	G	9	0.19	0.01	2.29E-98
rs3918226	T	7	0.13	0.02	1.69E-09
rs4420638	G	19	0.09	0.01	7.07E-11
rs4468572	C	15	0.08	0.01	4.44E-16
rs4593108	G	4	-0.07	0.01	8.82E-10
rs515135	C	2	0.07	0.01	3.09E-08
rs55730499	T	6	0.32	0.02	5.39E-39
rs56062135	T	15	-0.07	0.01	4.52E-09
rs56289821	A	19	-0.13	0.02	4.44E-15
rs56336142	C	6	-0.07	0.01	1.85E-08
rs663129	A	18	0.06	0.01	3.20E-08
rs6689306	G	1	-0.06	0.01	2.60E-09
rs67180937	G	1	0.08	0.01	1.01E-12
rs7212798	C	17	0.08	0.01	1.88E-08
rs7528419	G	1	-0.11	0.01	1.97E-23
rs7568458	A	2	0.06	0.01	3.62E-10
rs8042271	A	15	-0.10	0.02	3.68E-08
rs9349379	G	6	0.13	0.01	1.81E-42
rs9970807	T	1	-0.13	0.02	5.00E-14

Supplemental Table 4. Genetic instruments for diabetes in the bi-directional MR analysis

SNP	effect_allele	chromosome	beta	se	pval
rs10077431	A	5	-0.05	0.009	4.75E-08
rs10087241	A	8	-0.05	0.008	2.80E-09
rs10100265	C	8	-0.05	0.008	6.29E-10
rs10114341	C	9	-0.04	0.007	1.15E-08
rs10401969	C	19	0.09	0.013	4.13E-12
rs1050226	G	6	-0.05	0.007	3.34E-11
rs1061813	A	5	-0.04	0.007	3.37E-09
rs1063355	G	6	0.07	0.008	3.72E-19
rs10740322	A	10	0.05	0.009	2.11E-08
rs10811661	C	9	-0.16	0.010	4.13E-58
rs10830963	G	11	0.09	0.008	5.85E-30
rs10842994	T	12	-0.08	0.009	1.02E-16
rs10974438	C	9	0.06	0.008	3.01E-15
rs11098676	C	4	0.05	0.010	2.03E-08
rs11107116	T	12	0.05	0.009	3.75E-08
rs1111875	T	10	-0.09	0.007	3.61E-39
rs11257655	T	10	0.07	0.009	1.97E-17
rs1127655	T	1	-0.04	0.008	2.47E-08
rs11708067	G	3	-0.10	0.009	5.93E-29
rs11925227	A	3	-0.05	0.010	2.25E-08
rs11926707	C	3	0.05	0.008	1.69E-08
rs12088739	G	1	-0.09	0.013	9.79E-12
rs12299509	G	12	0.05	0.007	2.09E-10
rs12617659	T	2	-0.07	0.010	2.83E-11
rs12910825	G	15	0.05	0.007	2.16E-12
rs12945601	C	17	-0.05	0.008	1.72E-09
rs12970134	A	18	0.06	0.008	5.31E-12
rs13234269	A	7	-0.06	0.008	6.98E-14
rs13239186	T	7	0.05	0.009	2.70E-10
rs13330951	G	16	-0.05	0.008	1.54E-08
rs13389219	T	2	-0.07	0.007	2.11E-22
rs1359790	A	13	-0.08	0.008	2.80E-23
rs1496653	G	3	-0.08	0.009	2.57E-18
rs1552224	C	11	-0.10	0.010	8.64E-25
rs16988333	G	22	-0.07	0.013	9.17E-09
rs17086692	T	4	-0.05	0.008	2.48E-08
rs17168486	T	7	0.07	0.009	2.18E-15
rs17405722	A	17	0.09	0.015	2.28E-09
rs17411031	G	8	-0.05	0.008	3.04E-08
rs1758632	G	9	0.05	0.008	1.36E-09
rs17631783	T	17	-0.05	0.009	3.95E-08
rs17791513	G	9	-0.10	0.015	4.61E-12
rs1801214	T	4	0.09	0.007	5.52E-34
rs1899951	T	3	-0.11	0.011	1.64E-24
rs2058913	T	15	-0.05	0.008	3.26E-10
rs2237892	T	11	-0.10	0.016	8.75E-10
rs2246618	T	6	0.05	0.008	1.20E-09

rs2261181	T	12	0.10	0.012	9.18E-17
rs2294120	G	8	-0.04	0.008	1.62E-08
rs2296173	G	1	0.07	0.009	7.66E-14
rs2299383	T	7	0.04	0.007	1.49E-08
rs243019	C	2	0.06	0.007	2.29E-15
rs2493394	G	1	0.07	0.011	1.15E-10
rs2796441	A	9	-0.07	0.007	1.96E-22
rs2820426	G	1	0.05	0.007	1.30E-12
rs2867125	C	2	0.06	0.010	4.33E-10
rs2908282	A	7	0.06	0.009	4.25E-09
rs2925979	C	16	-0.05	0.008	9.06E-12
rs2943656	G	2	0.09	0.007	6.70E-34
rs3217992	T	9	0.05	0.007	7.23E-13
rs340874	C	1	0.06	0.007	8.41E-18
rs348330	A	1	-0.05	0.008	1.86E-09
rs3756784	G	6	0.05	0.009	2.59E-08
rs3802177	A	8	-0.12	0.008	2.32E-52
rs459193	G	5	0.07	0.008	8.81E-18
rs4622883	G	3	-0.04	0.008	3.02E-08
rs4686471	C	3	0.05	0.008	4.28E-11
rs4812829	A	20	0.05	0.010	2.44E-08
rs4823182	G	22	0.05	0.008	3.36E-10
rs4865796	A	5	0.05	0.008	1.33E-11
rs516946	C	8	0.08	0.009	3.16E-22
rs5215	T	11	-0.07	0.007	2.09E-20
rs55966194	G	20	-0.05	0.009	2.25E-09
rs576674	A	13	-0.07	0.010	1.79E-11
rs6059662	G	20	0.04	0.008	1.51E-08
rs61953351	T	12	-0.07	0.009	1.98E-14
rs622217	C	6	-0.05	0.008	3.13E-10
rs6494307	G	15	-0.04	0.008	1.67E-08
rs6515236	C	20	-0.05	0.009	3.34E-08
rs67232546	T	11	0.06	0.010	4.66E-10
rs6767484	G	3	0.12	0.008	2.70E-56
rs6785040	C	3	-0.06	0.011	1.26E-08
rs6795735	T	3	-0.06	0.007	1.63E-14
rs6878122	A	5	-0.06	0.008	1.19E-12
rs6960043	C	7	0.06	0.007	3.61E-19
rs7144011	T	14	0.05	0.009	1.64E-08
rs7177055	A	15	0.06	0.008	2.75E-16
rs7240767	C	18	0.05	0.008	2.16E-08
rs72802358	C	16	-0.12	0.013	1.97E-18
rs72892910	T	6	0.06	0.010	6.43E-11
rs735949	C	4	-0.07	0.011	1.95E-11
rs753270	C	10	0.05	0.008	2.70E-11
rs7561798	G	2	0.04	0.007	2.79E-08
rs7572970	G	2	0.06	0.009	1.39E-11
rs7607777	T	2	-0.14	0.013	9.40E-28
rs7674212	T	4	-0.05	0.008	6.18E-10

rs7685296	T	4	-0.05	0.008	2.32E-10
rs7729395	T	5	0.14	0.016	1.10E-17
rs7756992	G	6	0.13	0.008	6.00E-62
rs7786095	G	7	-0.07	0.013	9.64E-09
rs780094	C	2	0.07	0.007	5.16E-21
rs7845219	C	8	-0.04	0.007	4.54E-09
rs7903146	T	10	0.31	0.008	########
rs7929543	C	11	0.08	0.014	2.20E-09
rs7955901	T	12	-0.04	0.007	7.16E-10
rs8068804	A	17	0.06	0.008	4.41E-14
rs8108269	G	19	0.06	0.008	3.11E-16
rs825476	T	12	0.05	0.007	6.80E-13
rs840967	A	2	-0.05	0.008	5.44E-10
rs849135	A	7	-0.10	0.007	1.04E-43
rs853974	C	6	-0.06	0.009	7.86E-12
rs9369425	A	6	-0.05	0.009	1.13E-10
rs963740	T	13	-0.05	0.009	2.23E-08
rs9844972	C	3	0.10	0.015	1.03E-10
rs9894220	G	17	-0.06	0.008	1.52E-13
rs9928094	G	16	0.10	0.007	3.59E-47
rs993380	G	4	-0.05	0.008	4.59E-10
rs9940149	A	16	-0.06	0.010	9.29E-10

Supplemental Table 5. Genetic instruments for each protein included in the berberine signature identified based on the GWAS of proteins in men in UK Biobank

Protein	SNP	effect_allele	beta	se	pval	cis-SNP (Y or N)
APOM	rs111680800	A	-0.07	0.01	2.63E-16	N
APOM	rs1260326	C	-0.07	0.01	4.83E-17	N
APOM	rs17231506	T	0.11	0.01	4.22E-35	N
APOM	rs1800777	A	-0.13	0.02	2.67E-09	N
APOM	rs1800961	T	-0.22	0.02	1.60E-20	N
APOM	rs3130623	T	0.09	0.01	1.97E-18	Y
APOM	rs34931250	T	0.12	0.02	2.12E-11	N
APOM	rs429358	C	0.07	0.01	1.97E-10	N
APOM	rs56332871	A	0.05	0.01	2.24E-08	N
APOM	rs56414040	A	-0.13	0.02	1.34E-15	N
APOM	rs59473078	T	-0.21	0.03	4.85E-11	N
APOM	rs6129727	A	0.05	0.01	1.10E-08	N
APOM	rs6929090	A	-0.14	0.03	3.53E-08	N
APOM	rs6999158	A	0.06	0.01	4.65E-10	N
APOM	rs738408	T	-0.07	0.01	3.87E-13	N
APOM	rs805270	G	-0.72	0.02	3.30E-205	Y
APOM	rs9260332	G	-0.26	0.05	1.09E-08	N
APOM	rs9265867	T	-0.07	0.01	1.59E-09	N
APOM	rs9414801	A	0.05	0.01	8.28E-09	N
CA1	rs35959442	G	-0.08	0.01	1.19E-15	N
CA1	rs3811444	T	0.08	0.01	1.66E-18	N
CA1	rs7175720	C	-0.13	0.01	4.38E-34	N
CA1	rs7824715	G	-0.08	0.01	1.38E-18	Y
F11	rs115668102	G	0.14	0.02	9.68E-12	N
F11	rs12186258	A	0.11	0.01	1.58E-31	Y
F11	rs1260326	C	-0.05	0.01	1.90E-09	N
F11	rs12651524	T	-0.06	0.01	3.04E-08	N
F11	rs140645146	T	-0.28	0.04	6.30E-15	N
F11	rs1846899	T	-0.21	0.01	6.96E-46	N
F11	rs266770	G	-0.09	0.01	2.40E-13	N
F11	rs28576278	G	0.21	0.03	8.14E-10	Y
F11	rs35799453	C	0.20	0.03	1.06E-09	N
F11	rs4253417	C	0.36	0.01	0	Y
F11	rs5030062	C	0.44	0.01	0	N
F11	rs622871	G	0.06	0.01	9.26E-10	N
F11	rs6804147	G	0.09	0.01	3.72E-12	N
F11	rs6838463	A	-0.08	0.01	1.59E-08	Y
F11	rs75105010	A	0.23	0.03	3.29E-11	Y
F11	rs75357674	G	-0.13	0.02	1.09E-11	N
F11	rs7625893	T	0.10	0.02	1.68E-08	N
F11	rs7685922	T	-0.09	0.01	1.27E-17	N
F11	rs77188638	A	0.42	0.02	1.38E-72	N
REG1A	rs11200615	G	0.11	0.02	9.30E-09	N
REG1A	rs116344400	T	0.17	0.03	6.99E-11	Y
REG1A	rs283851	C	0.29	0.02	3.63E-73	Y
REG1A	rs3013223	G	0.15	0.01	4.45E-70	N

REG1A	rs3019500	T	-0.07	0.01	7.56E-15	N
REG1A	rs3754810	T	-0.28	0.02	7.85E-45	Y
REG1A	rs417144	T	-0.09	0.01	4.10E-24	Y
REG1A	rs529565	C	0.13	0.01	1.48E-46	N
REG1A	rs535777	C	0.07	0.01	2.01E-09	N
REG1A	rs601338	A	-0.15	0.01	6.80E-69	N
REG1A	rs708686	T	0.10	0.01	1.50E-27	N
REG1A	rs72816652	T	0.10	0.01	9.29E-11	Y
REG1A	rs75924779	A	0.25	0.03	5.63E-15	N
REG1A	rs76841471	G	0.42	0.02	2.48E-138	Y
REG1A	rs77541989	A	0.18	0.03	8.63E-11	N
PRSS2	rs17032925	C	-0.09	0.02	6.63E-09	N
PRSS2	rs17269	G	0.06	0.01	3.29E-10	Y
PRSS2	rs174554	G	0.05	0.01	2.89E-09	N
PRSS2	rs1936812	G	-0.05	0.01	1.98E-08	N
PRSS2	rs2230033	A	0.05	0.01	3.38E-08	N
PRSS2	rs2277710	C	0.11	0.02	2.34E-11	N
PRSS2	rs2534792	A	0.06	0.01	5.66E-09	N
PRSS2	rs34146038	A	0.07	0.01	9.48E-11	N
PRSS2	rs3752404	G	-0.12	0.01	6.47E-42	Y
PRSS2	rs4733612	A	0.07	0.01	5.41E-14	N
PRSS2	rs4987697	T	-0.10	0.02	1.10E-08	Y
PRSS2	rs686056	A	-0.08	0.01	1.76E-17	N
PRSS2	rs7037712	A	-0.08	0.01	9.97E-09	N
PRSS2	rs72802342	A	-0.25	0.02	3.37E-48	N
PRSS2	rs7404039	G	-0.11	0.01	8.31E-37	N
PRSS2	rs7833694	C	0.06	0.01	7.11E-11	N
PRSS2	rs9375435	T	-0.07	0.01	8.44E-15	N
PRSS2	rs9673342	C	0.08	0.01	2.08E-15	N
MBL2	rs10824739	T	-0.09	0.01	1.04E-20	N
MBL2	rs10824769	C	0.40	0.01	0	Y
MBL2	rs10824843	G	-0.07	0.01	1.32E-12	N
MBL2	rs11003139	T	0.31	0.01	1.06E-140	Y
MBL2	rs117353012	A	0.35	0.03	8.31E-32	N
MBL2	rs117804300	A	0.15	0.02	1.39E-09	N
MBL2	rs117897064	T	0.32	0.03	6.88E-22	N
MBL2	rs1260326	C	0.06	0.01	2.63E-20	N
MBL2	rs149901071	G	0.54	0.05	2.37E-31	N
MBL2	rs1800451	T	-1.06	0.03	2.94E-257	Y
MBL2	rs2001945	C	0.05	0.01	6.05E-12	N
MBL2	rs2157780	G	-0.04	0.01	1.95E-08	N
MBL2	rs36124964	C	-0.04	0.01	2.65E-08	N
MBL2	rs505922	C	0.22	0.01	3.72E-203	N
MBL2	rs55714260	G	-0.41	0.03	1.36E-43	Y
MBL2	rs57078070	T	-0.59	0.01	0	Y
MBL2	rs61859934	A	-0.19	0.03	6.19E-12	N
MBL2	rs72789185	T	0.17	0.03	2.80E-10	N
MBL2	rs72798652	G	-0.36	0.03	1.04E-28	N
MBL2	rs72800342	C	0.12	0.02	9.05E-14	N

MBL2	rs73341311	T	0.31	0.02	1.70E-39	Y
MBL2	rs7905367	C	-0.07	0.01	8.92E-10	N
MBL2	rs79778313	G	0.11	0.01	2.44E-15	N
MBL2	rs8048539	C	-0.04	0.01	2.47E-08	N
CCL5	rs10740118	C	0.11	0.01	3.72E-34	N
CCL5	rs12027184	G	-0.06	0.01	1.43E-08	N
CCL5	rs13236689	G	0.05	0.01	3.82E-10	N
CCL5	rs1354034	C	-0.07	0.01	5.95E-15	N
CCL5	rs2107538	T	-0.21	0.01	2.07E-80	Y
CCL5	rs2189336	A	-0.05	0.01	2.60E-08	N
CCL5	rs2576570	C	0.06	0.01	1.26E-10	N
CCL5	rs3827978	T	0.06	0.01	4.33E-11	N
CCL5	rs4572884	T	-0.05	0.01	1.00E-09	N
CCL5	rs61978213	A	0.12	0.02	1.62E-08	N
CCL5	rs6427756	G	-0.05	0.01	3.63E-08	N
CCL5	rs6482043	G	0.06	0.01	8.16E-12	N
CCL5	rs6993770	T	-0.07	0.01	1.32E-12	N
CCL5	rs892090	G	0.10	0.01	4.19E-17	N
CD46	rs115478735	T	-0.06	0.01	3.71E-08	N
CD46	rs1238357	T	0.08	0.01	5.70E-19	N
CD46	rs859705	G	-0.09	0.01	6.12E-23	Y
CD46	rs9468900	A	-0.06	0.01	3.33E-09	N
CA4	rs112515515	T	0.05	0.01	2.04E-09	N
CA4	rs117154637	T	-0.77	0.02	2.29E-247	Y
CA4	rs117742403	A	-0.39	0.04	1.58E-23	N
CA4	rs12975366	C	-0.06	0.01	3.85E-12	N
CA4	rs147414880	T	-0.39	0.03	2.89E-30	Y
CA4	rs4760	G	-0.16	0.01	2.08E-42	N
CA4	rs687289	A	0.16	0.01	2.33E-71	N
CA4	rs7216271	T	0.15	0.01	2.47E-25	Y
CA4	rs78058190	A	-0.22	0.02	1.58E-18	N
THBS4	rs10251492	A	0.07	0.01	9.07E-15	N
THBS4	rs116172614	G	0.15	0.02	4.45E-11	Y
THBS4	rs11661117	G	-0.05	0.01	4.63E-08	N
THBS4	rs117163080	C	0.08	0.01	1.13E-10	N
THBS4	rs1257218	C	-0.06	0.01	1.80E-09	N
THBS4	rs17472293	A	0.08	0.01	1.53E-19	N
THBS4	rs17710	T	-0.08	0.01	3.23E-10	N
THBS4	rs182042	A	-0.13	0.01	2.45E-42	N
THBS4	rs1926736	G	0.09	0.01	2.92E-26	N
THBS4	rs2099651	A	-0.06	0.01	3.29E-11	Y
THBS4	rs256438	G	0.21	0.01	7.60E-123	Y
THBS4	rs34333163	G	0.09	0.02	2.21E-08	N
THBS4	rs55714927	T	0.16	0.01	5.01E-48	N
THBS4	rs597808	G	0.06	0.01	2.53E-12	N
THBS4	rs687289	A	0.06	0.01	3.14E-12	N
THBS4	rs73225851	T	-0.11	0.02	3.54E-08	N
THBS4	rs73632737	C	-0.13	0.02	1.46E-17	N
COMP	rs10422974	C	-0.06	0.01	4.47E-09	N

COMP	rs1049130	G	0.05	0.01	2.57E-10	N
COMP	rs11237459	C	-0.06	0.01	3.90E-08	N
COMP	rs112771035	G	-0.11	0.02	1.45E-11	N
COMP	rs12974746	G	-0.54	0.03	2.12E-85	Y
COMP	rs17750957	C	0.09	0.01	3.04E-16	Y
COMP	rs3184504	C	0.07	0.01	2.40E-14	N
COMP	rs369705328	C	0.13	0.01	3.90E-23	N
COMP	rs4233367	C	-0.06	0.01	8.99E-12	N
COMP	rs55714927	T	0.10	0.01	4.60E-20	N
COMP	rs56278466	G	0.12	0.01	2.37E-38	N
COMP	rs7250885	G	0.16	0.01	1.41E-32	Y
COMP	rs7294354	G	-0.05	0.01	4.04E-08	N
DEFA1_DEFA1B	rs10103048	C	-0.05	0.01	2.29E-08	N
DEFA1_DEFA1B	rs10411858	A	-0.06	0.01	1.75E-08	N
DEFA1_DEFA1B	rs10774625	G	-0.06	0.01	7.21E-12	N
DEFA1_DEFA1B	rs10872428	T	-0.10	0.01	4.41E-21	N
DEFA1_DEFA1B	rs11137077	T	0.14	0.02	8.53E-11	N
DEFA1_DEFA1B	rs112043702	T	0.13	0.02	4.76E-10	N
DEFA1_DEFA1B	rs117582969	C	0.44	0.04	1.37E-35	N
DEFA1_DEFA1B	rs12783472	G	-0.06	0.01	1.20E-08	N
DEFA1_DEFA1B	rs149286471	C	-0.27	0.01	1.04E-122	N
DEFA1_DEFA1B	rs2615787	G	-0.09	0.01	1.67E-23	N
DEFA1_DEFA1B	rs2951863	C	-0.10	0.01	1.96E-28	N
DEFA1_DEFA1B	rs3128959	A	0.17	0.01	1.36E-35	N
DEFA1_DEFA1B	rs34914251	C	0.38	0.03	8.82E-37	N
DEFA1_DEFA1B	rs35763146	A	-0.16	0.02	8.31E-14	N
DEFA1_DEFA1B	rs3758139	G	0.12	0.02	2.88E-09	N
DEFA1_DEFA1B	rs3826331	C	-0.09	0.01	1.02E-27	N
DEFA1_DEFA1B	rs3917932	G	-0.07	0.01	1.36E-17	N
DEFA1_DEFA1B	rs61740288	A	0.23	0.03	1.35E-14	N
DEFA1_DEFA1B	rs6782812	A	-0.08	0.01	3.16E-08	N
DEFA1_DEFA1B	rs6988346	T	-0.10	0.01	1.40E-27	N
DEFA1_DEFA1B	rs74678278	A	-0.06	0.01	4.64E-09	N
DEFA1_DEFA1B	rs7846314	T	0.11	0.01	1.93E-22	N
LCN2	rs11670056	T	-0.10	0.02	1.10E-09	N
LCN2	rs12521723	T	0.09	0.01	8.78E-16	N
LCN2	rs1867839	T	-0.05	0.01	2.24E-08	N
LCN2	rs2158799	G	0.05	0.01	4.36E-08	N
LCN2	rs218264	T	0.05	0.01	4.60E-08	N
LCN2	rs2232624	A	-0.12	0.02	4.55E-10	Y
LCN2	rs2287104	C	-0.06	0.01	2.11E-09	N
LCN2	rs2502819	A	-0.26	0.05	1.98E-08	Y
LCN2	rs3014874	A	0.07	0.01	3.27E-12	N
LCN2	rs3917932	G	-0.07	0.01	3.12E-15	N
LCN2	rs3936197	A	-0.10	0.01	6.72E-33	N
LCN2	rs59697075	T	-0.06	0.01	2.41E-11	N
LCN2	rs62246445	A	0.07	0.01	2.10E-10	N
LCN2	rs6870370	C	0.07	0.01	1.11E-14	N
LCN2	rs7579665	C	-0.06	0.01	8.44E-09	N

LCN2	rs76641566	A	0.13	0.02	2.58E-15	N
LCN2	rs7846314	T	0.10	0.01	3.91E-22	N
LCN2	rs9830594	T	0.06	0.01	4.84E-09	N
TGFBR3	rs11207423	C	0.11	0.01	2.97E-27	N
TGFBR3	rs115004469	T	-0.15	0.03	3.45E-08	N
TGFBR3	rs11702316	A	0.09	0.01	8.37E-17	N
TGFBR3	rs12023393	G	-0.21	0.03	1.28E-13	Y
TGFBR3	rs1236213	T	-0.14	0.01	9.63E-55	N
TGFBR3	rs1477717	T	0.20	0.02	8.16E-18	N
TGFBR3	rs1805109	T	-0.22	0.02	2.25E-49	Y
TGFBR3	rs229048	G	0.13	0.01	1.33E-36	N
TGFBR3	rs2298657	C	0.18	0.03	1.99E-09	N
TGFBR3	rs2830885	A	-0.05	0.01	6.51E-10	N
TGFBR3	rs369705328	C	0.09	0.01	5.14E-11	N
TGFBR3	rs56278466	G	0.05	0.01	1.18E-09	N
TGFBR3	rs72704449	C	0.13	0.02	3.12E-09	N
TGFBR3	rs72712451	T	0.09	0.01	1.10E-19	Y
TGFBR3	rs73187807	T	0.25	0.03	4.19E-21	N
TGFBR3	rs74347297	G	0.19	0.03	3.08E-12	N
TGFBR3	rs7633214	A	-0.08	0.01	3.19E-09	N
PLA2G7	rs111617668	T	-0.11	0.02	7.77E-10	N
PLA2G7	rs112019714	C	0.22	0.03	4.35E-18	N
PLA2G7	rs13218408	T	-0.15	0.02	6.99E-17	Y
PLA2G7	rs1786140	C	0.07	0.01	6.63E-17	N
PLA2G7	rs2954021	G	-0.05	0.01	3.34E-08	N
PLA2G7	rs4803759	C	0.08	0.01	8.04E-16	N
PLA2G7	rs56210316	G	-0.16	0.02	7.19E-12	N
PLA2G7	rs57217136	C	-0.11	0.01	9.51E-18	N
PLA2G7	rs7412	T	-0.40	0.02	5.09E-144	N
PLA2G7	rs7528419	G	-0.10	0.01	7.87E-24	N
PLA2G7	rs769449	A	0.25	0.01	2.34E-85	N
PLA2G7	rs964184	C	-0.08	0.01	3.20E-12	N
LTBP2	rs1070073	G	0.07	0.01	3.20E-17	N
LTBP2	rs146607429	T	-0.23	0.03	3.19E-13	Y
LTBP2	rs2108759	A	0.07	0.01	1.95E-08	N
LTBP2	rs2691582	T	-0.04	0.01	1.55E-09	N
LTBP2	rs333947	A	-0.06	0.01	6.02E-09	N
LTBP2	rs3751198	G	0.08	0.01	1.23E-25	N
LTBP2	rs4899520	A	-0.06	0.01	8.53E-17	Y
LTBP2	rs4920605	A	0.08	0.01	9.55E-27	N
LTBP2	rs55707100	T	-0.13	0.02	4.97E-09	N
LTBP2	rs61804164	C	0.07	0.01	1.90E-08	N
LTBP2	rs703630	C	-0.06	0.01	9.26E-11	N
LTBP2	rs7107356	G	0.04	0.01	2.04E-09	N
LTBP2	rs72837687	A	0.06	0.01	2.66E-10	N
LTBP2	rs74384554	C	0.22	0.03	7.91E-13	Y
LTBP2	rs8007114	C	0.07	0.01	8.93E-09	N
LTBP2	rs8034829	C	-0.07	0.01	8.77E-09	N
LTBP2	rs888414	A	-0.09	0.01	5.03E-36	Y

FCN2	rs10119380	C	0.12	0.01	8.22E-40	Y
FCN2	rs10502187	T	-0.43	0.01	0	N
FCN2	rs10935473	T	-0.05	0.01	3.66E-11	N
FCN2	rs11103629	G	0.23	0.02	3.96E-42	N
FCN2	rs117341751	A	0.13	0.02	3.46E-08	N
FCN2	rs118141657	C	-0.32	0.04	8.66E-13	Y
FCN2	rs143334146	T	0.30	0.05	7.37E-10	Y
FCN2	rs146419026	A	-0.23	0.04	5.39E-10	N
FCN2	rs149085597	A	-0.40	0.05	1.05E-18	Y
FCN2	rs149742777	A	0.18	0.03	2.52E-08	N
FCN2	rs17039706	T	-0.06	0.01	1.32E-10	N
FCN2	rs30376	T	0.11	0.01	7.06E-37	N
FCN2	rs35677470	A	0.10	0.01	3.51E-12	N
FCN2	rs4418728	T	-0.04	0.01	2.24E-08	N
FCN2	rs4584234	A	-0.07	0.01	3.36E-13	N
FCN2	rs4842221	G	-0.09	0.01	2.48E-18	N
FCN2	rs55871208	C	0.33	0.01	7.13E-214	Y
FCN2	rs57464688	A	-0.60	0.02	3.83E-257	Y
FCN2	rs61804164	C	-0.10	0.01	1.58E-15	N
FCN2	rs62573360	A	-0.19	0.03	4.15E-09	N
FCN2	rs681094	G	0.09	0.01	3.65E-14	N
FCN2	rs7045964	G	0.25	0.03	2.92E-13	Y
FCN2	rs72550870	C	-0.22	0.02	1.02E-22	N
FCN2	rs72774480	T	-0.16	0.02	1.01E-12	Y
FCN2	rs72776330	C	-0.31	0.03	8.43E-34	Y
FCN2	rs7295693	C	0.05	0.01	2.27E-09	N
FCN2	rs73572021	A	-0.18	0.02	2.62E-16	Y
FCN2	rs7484541	T	-0.07	0.01	1.87E-15	N
FCN2	rs74912513	G	0.10	0.01	3.16E-11	N
FCN2	rs76804528	T	-0.19	0.03	3.60E-09	N
FCN2	rs77293080	G	-0.14	0.02	3.06E-08	N
FCN2	rs7869093	G	-0.12	0.01	1.70E-18	N
FCN2	rs7871721	T	-0.80	0.02	0	N
FCN2	rs9410002	A	-0.08	0.01	1.96E-19	N
CNDP1	rs117232418	C	-0.16	0.02	1.94E-11	N
CNDP1	rs12462045	A	0.16	0.01	6.42E-43	N
CNDP1	rs138923686	C	-0.23	0.04	2.94E-08	N
CNDP1	rs139622949	T	-0.40	0.05	1.35E-15	Y
CNDP1	rs147233090	T	-0.18	0.03	5.72E-11	N
CNDP1	rs1559807	G	0.06	0.01	2.68E-09	N
CNDP1	rs17010676	T	-0.22	0.04	4.17E-08	N
CNDP1	rs17817077	A	0.26	0.01	1.68E-190	Y
CNDP1	rs28929474	T	-0.24	0.03	2.91E-15	N
CNDP1	rs2954021	G	-0.06	0.01	5.21E-14	N
CNDP1	rs34560823	T	0.07	0.01	5.65E-11	N
CNDP1	rs4329999	A	-0.18	0.01	7.90E-74	Y
CNDP1	rs523118	G	0.09	0.01	5.41E-21	N
CNDP1	rs73114872	C	-0.06	0.01	1.76E-08	N

CNDP1	rs738409	G	-0.06	0.01	3.59E-10	N
CNDP1	rs8084058	G	0.08	0.01	9.30E-20	Y
CNDP1	rs8102710	T	-0.33	0.01	2.36E-236	N
CNDP1	rs9949990	T	-0.25	0.04	1.12E-10	Y
CNDP1	rs9953129	G	0.09	0.01	2.90E-22	Y

Supplemental Table 6. Proteins associated with IHD and/or diabetes

Outcome	Protein	coefficient	logOR	p.adj	MR showed consistent direction	possible mediator (Y/N)
IHD	PRSS2	-0.35	0.22	2.87E-10	Y	Y
IHD	SHBG	0.21	-0.11	1.64E-05	Y	Y
Diabetes	CNDP1	-0.18	0.23	5.32E-05	Y	Y
Diabetes	THBS4	-0.15	0.65	4.28E-34	Y	Y
Diabetes	LCN2	-0.02	0.29	9.90E-06	Y	Y
Diabetes	F11	-0.13	0.36	1.02E-05	Y	Y
Diabetes	CCL5	-0.23	0.11	2.26E-05	Y	Y
Diabetes	Testosterone	0.21	-0.52	1.51E-54	Y	Y
Diabetes	SHBG	0.21	-0.51	3.80E-39	Y	Y
IHD	TGFBR3	-0.11	-0.15	3.30E-02	Y	N
IHD	DEFA1	0.27	0.20	9.17E-08	Y	N
Diabetes	APOM	-0.25	-1.87	4.64E-101	Y	N
Diabetes	REG1A	0.27	0.45	6.43E-22	Y	N
Diabetes	PLA2G7	-0.14	-1.44	2.95E-80	Y	N
Diabetes	TGFBR3	-0.11	-0.35	3.11E-05	Y	N
Diabetes	DEFA1	0.27	0.15	1.43E-03	Y	N

logOR refers to log odds ratio in logistic regression; p.adj refers to adjusted p value.

Supplemental Table 7. Number of cis-SNPs for each protein

Protein	# of cis-SNP(s)
APOM	2
CA1	1
CA4	3
CCL5	1
CD46	1
CNDP1	6
COMP	3
F11	5
FCN2	10
LCN2	2
LTBP2	4
MBL2	6
PLA2G7	1
PRSS2	3
REG1A	6
TGFBR3	3
THBS4	3

Supplemental Table 8. The association of berberine score with ischemic heart disease and diabetes in observational study among 15,915 women in UK Biobank

Outcomes	OR	95% CI	P value
IHD	0.82	0.72, 0.92	0.001
Diabetes	0.64	0.55, 0.74	8.8×10^{-10}

*Adjusted for age, ethnicity, smoking, alcohol drinking, Townsend index, education, and physical activity.
